# C-peptide promotes myogenic differentiation in vitro and low serum levels are associated with sarcopenia in adults and the elderly

**DOI:** 10.1186/s12967-026-07983-9

**Published:** 2026-03-11

**Authors:** Yvelise Ferro, Alberto Castagna, Samantha Maurotti, Francesca Rita Noto, Elisa Mazza, Valeria Rizzo, Carmelo Pujia, Angelo Galluccio, Angela Sciacqua, Carmine Gazzaruso, Stefano Romeo, Arturo Pujia, Tiziana Montalcini

**Affiliations:** 1 Department of Medical and Surgical Sciences, University “Magna Graecia” of Catanzaro, 88100 Catanzaro, Italy; 2 Department of Clinical and Experimental Medicine, University “Magna Graecia” of Catanzaro, 88100 Catanzaro, Italy; 3 O.U. Clinical Nutrition, Renato Dulbecco Hospital, 88100 Catanzaro, Italy; 4 Department of Biomedical Sciences for Health, University of Milan, Milan, Italy; 5 Wallenberg Laboratory, Department of Molecular and Clinical Medicine, Institute of Medicine, The Sahlgrenska Academy, University of Gothenburg, Gothenburg, Sweden; 6 Department of Cardiology, Sahlgrenska University Hospital, Gothenburg, Sweden; 7 Department of Medicine, Huddinge Karolinska Institute, Stockholm, Sweden; 8 Research Center for the Prevention and Treatment of Metabolic Diseases, University “Magna Graecia”, 88100 Catanzaro, Italy

**Keywords:** Sarcopenia, Myogenic differentiation, C-peptide, Muscle atrophy, Fractures, Appendicular skeletal muscle mass

## Abstract

**Background:**

Sarcopenia, the age-related loss of muscle mass and function, is a growing public health problem. Its multifactorial causes including poor nutrition, physical inactivity, and underlying diseases. Research suggests a role for C-peptide in muscle regulation, but its link to sarcopenia is unclear. We hypothesized that low C-peptide associates with sarcopenia and fractures, and that it promotes myogenesis and protects muscle cells from atrophic damage in vitro.

**Methods:**

In this cross-sectional study, we analysed data from 191 individuals aged 50 or older, including serum C-peptide and appendicular skeletal muscle mass measurements, along with falls and fractures assessed at follow-up. Skeletal muscle parameters were evaluated using bioelectrical impedance analysis. In parallel, in vitro experiments were performed in C2C12 cells to investigate the effects of C-peptide on myogenic differentiation and its protective role against muscle damage.

**Results:**

Individuals in the lowest C-peptide tertile had significantly less appendicular skeletal muscle/sarcopenia than those in the highest (*p* < 0.001). Lower C-peptide significantly increased sarcopenia odds (OR: 0.34; *p* < 0.001). These individuals also showed higher falls (32%) and fractures (17%) rates compared to the highest tertile (14% and 0%). Fractures were linked to falls and low C-peptide levels (OR: 0.29; *p* = 0.01). In vitro, C-peptide promotes myogenic differentiation and reduced muscle damage in C2C12 myotubes.

**Conclusions:**

Our findings suggest the protective role of C-peptide in musculoskeletal health, demonstrating that higher levels are associated with a reduced risk of having low appendicular skeletal muscle mass/sarcopenia and fractures in adults and the elderly. Moreover, C-peptide enhances myogenic differentiation and protects against in vitro nutrient-deprivation muscle damage. Further studies are warranted to explore the therapeutic utility of C-peptide for the prevention of sarcopenia and fractures in adulthood and aging.

**Supplementary Information:**

The online version contains supplementary material available at 10.1186/s12967-026-07983-9.

## Introduction

Sarcopenia, a condition marked by the gradual loss of skeletal muscle mass and strength, has multifactorial causes including reduced physical activity, inadequate nutrition, chronic diseases, inflammation, degeneration of neuromuscular junctions, and the natural aging process [1]. Sarcopenia represents a significant public health concern, as its prevalence is constantly increasing with aging global population [2]. The prevalence of sarcopenia varies between studies, ranging 10% and 27%, depending on classifications and cut-off points [3]. The prevalence among individuals younger than 60 years of age ranges from 8% to 36%, while for individuals older than 60 years of age the prevalence ranges from 10% to 27% [3]. This condition is associated with a higher risk of falls, fractures, hospital admissions, disability and mortality [4]. Currently, no effective pharmacological treatments are available for managing sarcopenia [5]. Early diagnosis and the development of innovative therapies are crucial to minimizing adverse clinical outcomes and enhancing quality of life.

C-peptide is a short peptide that links the A and B chains of insulin within the proinsulin molecule. Synthesized in the pancreas, it is released into the bloodstream in equimolar amounts with insulin [6]. Once considered biologically inactive, C-peptide is now recognized for its role in the progression of diabetic complications, such as nephropathy, retinopathy, and peripheral neuropathy [7]. Recent research has also revealed that low serum C-peptide levels are associated with decreased muscle mass and sarcopenia in individuals with type 2 diabetes mellitus [8–10].

Furthermore, a preclinical study on murine models demonstrated that C-peptide administration protects skeletal muscle mass from atrophy induced by type 1 diabetes [11]. These findings suggest a potential role for C-peptide not only in the physiology of muscle mass loss, but also as a therapeutic option. Research on the relationship between C-peptide and sarcopenia as well as fractures in individuals without diabetes remains limited. We hypothesized that serum C-peptide levels would be associated with the presence of low skeletal muscle mass or sarcopenia in adulthood and aging, and that they would also predict fracture occurrence. Additionally, we hypothesized that an in vitro model of myotube development could clarify C-peptide’s potential role in the differentiation mechanism.

## Subjects and methods

### Subjects

In this cross-sectional study, the population consisted of 191 adults and elderly individuals of both genders who underwent consecutive medical visits at the Clinical Nutrition Unit of the “R. Dulbecco” University Hospital in Catanzaro, Italy, from January 2015 to October 2024. The study included individuals with at least one measurement of serum C-peptide and appendicular skeletal muscle mass (ASMM), as recorded in the outpatient database. The incidence of falls and fractures was prospectively evaluated at follow-up. The study protocol was approved by the Territorial Ethics Committee of the Calabria Region (5/2023/CE approved September, 2023).

All participants signed written informed consent. The investigation conforms to the ethical principles outlined in the Declaration of Helsinki. Since diabetes (both type 1 and type 2) is associated with change of the levels of peptide C and diabetes causes sarcopenia, we excluded diabetic patients. The diagnosis of diabetes was established based on the following criteria: antidiabetic drugs use or fasting blood glucose ≥ 126 mg/dL [12].

Furthermore, individuals with severe chronic renal disease, liver disease, malignancies, rheumatic diseases, physical disabilities, and patients with NYHA functional class ≥ II were excluded. We also excluded all individuals taking medications or dietary supplements that could affect muscle mass, such as glucocorticoids, thyroid, sexual and growth hormones, psychotropic medications, and any nutritional supplement as reported in their medical records. Dyslipidemia was defined by blood levels of total cholesterol exceeding 200 mg/dL and/or triglycerides above 200 mg/dL, or the use of medications to lower lipid levels. Hypertension was identified when systolic blood pressure was at least 130 mmHg and/or diastolic blood pressure) was 85 mmHg or higher, or in cases where antihypertensive treatments were being used [13]. Participants were classified as current smokers if they had smoked more than 100 cigarettes in their lifetime and continued to smoke daily or occasionally [14].

Treatment for hypertension, and hyperlipidemia was not considered an exclusion criterion.

### Anthropometric assessments, muscular strength, and sarcopenia assessment

Body weight was assessed on a calibrated digital scale (Tanita BC-418MA model) accurate to 0.1 kg, and standing height was measured with a stadiometer (seca 213 model) accurate to 0.1 cm. Body mass index (BMI) was calculated, and obesity was defined for a BMI ≥ 30 kg/m^2^ [12].

Hand-foot bioelectrical impedance analysis (BIA) was performed on all patients to assess the fat mass (FM), skeletal muscle mass (SM), and ASMM. Specifically, after an overnight fast, participants were evaluated post-micturition and were instructed to refrain from physical activity prior to the measurements [15]. All assessments were conducted in the same room under controlled environmental conditions, maintaining a neutral temperature and a consistent time of day [15]. Participants were positioned supine on a non-conductive surface, ensuring a relaxed posture and minimal contact between limbs to prevent interference with the measurements. Two surface electrodes were placed on the right hand and right foot, at the wrist and ankle, near the metacarpal and metatarsal regions. Once the electrodes were secured and connected to the bioelectrical impedance device, a low-intensity electrical current at 50 kHz was applied [15]. After a brief stabilization period, resistance (R), reactance (Xc), and phase angle (PhA) were recorded.

Thus, ASMM, defined as the sum of arm and leg muscle mass, was calculated using the equations implemented in the manufacturer’s software (Akern, Bodygram Plus), which are based on the validated equation proposed by Sergi et al. [16]. Specifically, ASMM was estimated according to the following formula:

ASMM (kg) = -3.964 + (0.227*RI) + (0.095*weight) + (1.384*sex) + (0.064*Xc) [R(2) = 0.92 and SEE = 1.14 kg] [16].

The criteria established by the European Working Group on Sarcopenia in Older People (EWGSOP2) [17] were adopted to diagnose sarcopenia based on BIA and handgrip dynamometer measurements. In particular, a low ASMM was defined with a cut-off value of less than 20 kg for men and less than 15 kg for women [17]. Handgrip strength (HGS) data of the dominant hand were assessed using a handgrip dynamometer (DynX^®^™ Akern srl, Florence, Italy). For each strength test, three maximal isometric contractions were performed, with each contraction lasting 3 s. The average of the three measures was used as the criterion score. According with EWGSOP2 criteria, low HGS in men was diagnosed at a cut-off value of < 27 kg for men and < 16 kg for women [17].

### Fracture and fall assessment

The prevalence of morphological vertebral fractures was determined using lateral thoracolumbar radiographs. A vertebral fracture was defined as a loss of height in any dimension (anterior, middle, or posterior) exceeding 20% relative to an adjacent normal vertebra or expected vertebral height at that level. Vertebral fractures were also identified by endplate deformities, lack of parallelism, and general appearance changes compared to neighbouring vertebrae [18]. Non-vertebral fractures were assessed through interviews, clinical records or telephone calls. The number of fractures per participant was estimated, excluding pathological or high-energy fractures (e.g., from major trauma or uncommon sites like fingers or skull).

In addition, participants were asked if they had a fall, and if so to recall the number of falls from the first visit. A fall was defined as any event in which a person inadvertently or unintentionally comes to rest on the ground or another lower level such as a chair, toilet, or bed [19].

### Physical activity assessment

Participants’ physical activity levels were evaluated using the validated NPAQ-short questionnaire [20]. Based on their responses, participants were divided into two groups: those participating in moderate to vigorous physical activity (MVPA) and those categorized as sedentary or engaging in light physical activity. MVPA included activities such as brisk walking, dancing, gardening, sports or exercise, and walking domestic animals [21]. The classification method followed the approach outlined by Lee et al. [22].

### Biochemical evaluation

After an overnight fast, venous blood was collected into vacutainer tubes (Becton & Dickinson). Samples were allowed to clot at room temperature and were processed within 2 h. Serum was separated and analyzed immediately without freezing. Serum glucose, total cholesterol (TC), insulin, creatinine and high sensitive – C reactive protein (HS-CRP) were assessed using a chemiluminescent immunoassay according to the manufacturer’s instructions on a COBAS 8000 (Roche, Switzerland). According to Nkuna et al., to minimize pre-analytical variability and ensure the reproducibility of C-peptide measurements, analyses should be performed on fresh serum without freezing or the use of protease inhibitors [23]. In our study, specimens were centrifuged at > 2500 × g for 10 min at room temperature, and serum value of C-peptide was measured with a standard assay using chemiluminescent technology (ADVIA Centaur and ADVIA CentaurXP systems, Siemens Healthcare Diagnostic, USA, manufacturing by Kyowa Medex Co. Ltd, Japan.). According to the manufacturer’s specifications, the analytical sensitivity of this assay is approximately 0.05 ng/mL, corresponding to the lowest concentration of C peptide that can be reliably detected under standard conditions. Quality control assessments were performed daily for all measurements.

### Dietary intake assessment

A food frequency questionnaire (FFQ) was used to collect data on the frequency and portion sizes of foods and beverages consumed over the previous month [24]. The FFQ was administered by a trained and experienced dietitian. To address known biases in FFQs and enhance internal calibration, a less biased short-term instrument, a 24-hour recall (24 h), was also employed. The FFQ integrated portion size information with consumption frequency, asking participants to estimate their usual intake in specific units [24]. Portion sizes were based on typical servings (e.g., an egg or a slice of bread), and when no standard size was evident, commonly recognized measurements (such as a cup) were used. To improve accuracy, the questionnaire included images of portion sizes. All collected data were linked to a nutrient composition database (MetaDieta 3.0.1, San Benedetto del Tronto, Italy), which provided information on nutrient intake. Energy intake calculations were based on the National Institute of Food Research (INRAN) database from 2000, the European Institute of Oncology (IEO) databases from 2008 to 2015, and the Research Centre for Food and Nutrition (CREA) database from 2019. The MetaDieta 3.0.1 database includes information on over 6,500 food products and up to 150 food components, with annual updates.

### In vitro study

#### Cell cultures and maintenance

The mouse myoblast C2C12 cell line was purchased from Sigma-Aldrich (ACC No. 91031101, Lot No. 16k047). C2C12 cells were cultured in 100 mm tissue culture disks (Corning, Kennebunk, USA) and maintained in Dulbecco’s Modified Eagle Medium High Glucose (DMEM) (Corning, Kennebunk, USA) growth medium (GM), supplemented with 10% Fetal Bovine Serum (FBS) (SIAL, Rome, Italy) and 1% penicillin-streptomycin (100 µg/ml) (P/S) (SIAL, Rome, Italy) at 37 °C under a 5% CO_2_humidified atmosphere. Cells were sub-cultured when they reached 70 to 80% of confluence. Only cells between passages 3–10 were used in this study. Cells were routinely tested for mycoplasma assay.

#### Differentiation from myoblasts C2C12 to post-mitotic myotubes

C2C12 cells were cultured in p60 (Corning, Kennebunk, USA) at a density of 200,000 cells. After reaching 80–90% confluence (approximately 48 h), C2C12 cells were washed with phosphate-buffered saline (PBS) (Corning, Kennebunk, USA) and the GM was replaced with a differentiation medium (DMEM high glucose) containing 2% horse serum (HS) (Gibco by ThermoFisher Scientific). Furthermore, myoblasts were induced to differentiate with the following treatments: (a) rat C-peptide-2 (AS-63698, Anaspec) at a dose of 10 nM; (b) Insulin (I9278; Sigma Aldrich) at a dose of 2.5 nM; and (c) rats C-peptide-2 10 nM plus Insulin (2.5 nM) together. Doses of C-Peptide and Insulin (4:1 ratio) mimicking human physiology were used [25].

#### Induction of sarcopenia

To induce in vitro sarcopenia, C2C12 myotubes differentiated for 12 days were exposed to two atrophic conditions: nutrient deprivation and glucocorticoid excess [26]. Nutrient-deprivation–induced atrophy was generated by treating the myotubes with phosphate-buffered saline (PBS) for 4 h, while glucocorticoid-mediated atrophy was induced using dexamethasone (DEX, 100 µM) for 48 h. In both models, cells were treated with rat C-peptide-2 (10 nM), insulin (2.5 nM), or their combination. All treatments were performed in serum-free medium unless otherwise specified.

#### High-performance liquid chromatograph analysis

To evaluate whether C-peptide was utilized by the cells, its concentration in the culture medium was quantified before treatment and after 48 h of incubation using high-performance liquid chromatography (HPLC). HPLC analyses were performed using a Thermo Fisher Scientific Vanquish System Base equipped with a quaternary pump, split sampler, thermostated column compartment, and a UV/VIS variable-wavelength detector (Thermo Fisher Scientific, Rosano, Milan, Italy). The chromatographic method was optimized according to the physicochemical properties of C-peptide. Separation was achieved on a reversed-phase C18 analytical column (100 × 4.6 mm, 5 μm particle size) coupled to a compatible C18 guard column (Phenomenex, 4 × 3 mm). The mobile phase consisted of solvent A (water containing 0.1% trifluoroacetic acid, TFA) and solvent B (acetonitrile containing 0.1% TFA). Elution was performed at a constant flow rate of 1.0 mL/min using a linear gradient from 10% to 50% solvent B over 8 min. The total run time for each analysis was 15 min. UV detection was carried out at 214 and 220 nm, with a data acquisition rate of 2.0 Hz, a response time of 2 s, and a peak width of 0.2 min. Quantitative determination of C-peptide was performed using external calibration curves over a concentration range of 0.625–20 nM, showing excellent linearity (r² > 0.997; Supplemental Fig. 1. Data acquisition and processing were carried out using Chromeleon^®^ software (version 7.2).

#### Hematoxylin and eosin staining for measurement of myotube diameters

C2C12 myotubes were first washed twice with PBS and then fixed in 4% PFA for 10 min. Subsequently, the myotubes were stained with hematoxylin and eosin (H&E) solution (BioOptica) for 1 min at room temperature and observed using an optical microscope (Leica, Wetzlar, Germany). For each condition, six images were randomly captured from each well of the six-well plates using a Leica DM4 B Upright Microscope (Leica Microsystems), using an HC FL PLAN 40x/0.65 objective (Corning, Kennebunk, USA). For a selected portion of the image, a higher-magnification view was obtained using the same microscope, corresponding to a 25 μm scale. The diameters of three different sites in each myotube were measured using ImageJ software.

#### Protein extraction and western blot analysis

C2C12 protein lysates were prepared using M-PER protein extraction reagent (ThermoFisher Scientific) supplemented with protease (Roche, Mannheim, Germany) and phosphatase inhibitor cocktails (ThermoFisher Scientific), and briefly homogenized. Samples were loaded into 10% polyacrylamide gels with the PAGE system (BioRad, USA) and run in the SDS running buffer (25 mM Tris, 192 mM glycine, 0.1% SDS, pH 8.8). The run was monitored following ladders: Biorad Precision Plus Protein Standards #1,610,394. Afterward, proteins were transferred to TransBlot ^®^ Turbo™ Midi-Size Nitrocellulose (BioRad, USA) with Trans-Blot^®^ Turbo™ Transfer System (BioRad, USA) in TransBlot^®^ Turbo™ 5x transfer buffer (BioRad, USA). Membranes were blocked in 5% BSA powder in TBST (25 mM Tris, 150 mM NaCl, 0.2% Tween-20 (Sigma), pH 7.4 adjusted with HCl) and incubated overnight with the indicated primary antibodies dissolved in TBST containing 5% BSA. Rabbit-Anti phospho-p44/42 (Cell signaling, 1:1000), Rabbit-Anti phospho-Ampk (Cell signaling, 1:1000), Rabbit-Anti MyoD1 (Invitrogen, 1:1000), Mouse anti-MyHC (Merck Millipore Ltd, Ireland; dilution 1:1000), Mouse anti-Myogenin (Invitrogen, MA5-11486 dilution 1:1000) and mouse anti-β-Actin (Sigma-Aldrich, St. Louis; dilution 1:10000), primary antibodies were used. Blots were probed with primary antibodies, followed by the appropriate horseradish peroxidase (HRP)-conjugated secondary antibody (Bio-Rad, USA), and developed using Clarity™ Western ECL substrate (BioRad, USA). The image captures and densitometric analyses were performed with the ChemiDoc MP Imaging system (BioRad, USA) and ImageJ software, respectively.

#### RNA extraction and quantitative reverse transcriptase-PCR

Total RNA from C2C12 cells after treatment with C-peptide or insulin or both, was extracted using TRIzol reagent (Thermo Fisher Scientific) following the manufacturer’s instructions. cDNA was synthesized from 1 µg of total RNA using a high-capacity cDNA reverse transcription kit (Applied Biosystems by Thermo Fisher Scientific). The mRNA expression of *Bone Morphogenetic Protein 4* (*Bmp4*) (FW: GCTACCAGGCCTTCTACTGC and RW: ACTAGGGTCTGCACAATGGC) *Myosin heavy chain type I* (*MyHCI*) (FW: GAATGGCAAGACGGTGACTGTG and RW: GGAAGCGTAGCGCTCCTTGAG), *Myosin heavy chain type IIa* (*MyHCIIa*) (FW: ATCAACCAGCAGCTGGACACCA and RW: TCCAGCACGAACATGTGGTGGT), *Myosin heavy chain type IIx* (*MyHCIIx*) (FW: CCAATGAAACCAAGACTCCTGG and RW: TGCTATCGATGAACTGTCCCTC) *and Paired Box 7* (*Pax-7*) (FW: CAGTGTGCCATCTACCCATGCTTA and RW: GGTGCTTGGTTCAAATTGAGCC) were quantified through Real-Time PCR using SYBR^®^ Green dye (BioRad, USA). Data were analyzed using the 2^−∆∆Ct^ method and normalized to *β*-*Actin* (*ACTB*), whose expression stability was verified by low Ct variability across samples (SD < 0.50) [27].

### Statistical analysis

Data are presented as mean ± standard deviation (SD). Considering a prevalence of sarcopenia between 27% and 30%, to identify a difference of at least 25% in the prevalence of low ASMM/sarcopenia between the lowest and highest C-peptide tertiles, a sample size of at least 60 participants per group was required, assuming 80% power and a two-sided significance level of 0.05.

Pearson’s correlation analysis was performed, stratified by gender, to identify the variables correlated with ASMM, given that the continuous variables were normally distributed. Linear regression was performed to evaluate the association between C-peptide levels, all potential confounders identified to the Pearson’s correlation (i.e. age, BMI, FM, serum concentrations of C-peptide and insulin). Although both FM and BMI are correlated with ASMM, FM was used instead of BMI in the regression analysis, as it provides a more accurate estimate of the relationship between adipose tissue and muscle mass. Furthermore, we performed a binary logistic regression analysis to estimate the odds ratio (OR) of having low ASMM/sarcopenia based on potential confounding factors (as independent variables). In this analysis, low ASMM/sarcopenia was classified as a dependent binary outcome. Potential confounders included all variables that were correlated with ASMM in the univariate analysis with a *P* < 0.1.

Serum C-peptide levels were categorized into tertiles (1–3) to provide as sensitive indicators for assessing the association with ASMM. General characteristics of study participants across tertiles of C-peptide were examined using analysis of variance (ANOVA) for continuous variables or χ^2^ test for categorical variables. Two separate analyses were then performed: first, excluding individuals with sarcopenia to evaluate associations with isolated low ASMM; second, including both individuals with isolated low ASMM and those meeting criteria for sarcopenia to assess associations with the broader clinical phenotype. Adjusted prevalence of low ASMM and low ASMM/sarcopenia across tertiles of C-peptide was calculated using analysis of covariance (ANCOVA), adjusting for the variables that showed significant differences between tertiles according to ANOVA and χ^2^ tests (i.e. age, gender, FM and serum levels of glucose and insulin).

ROC curve analysis was conducted to identify the optimal C-peptide cut-off value for detecting low ASMM/sarcopenia.

Furthermore, we assessed the prevalence of falls and fractures at follow-up across C-peptide tertiles using the χ^2^ test. We also performed a logistic regression analysis to estimate the odds of experiencing falls and fractures at follow-up according to C-peptide tertiles. Finally, a ROC curve analysis was performed to determine the optimal cut-off value of C-peptide to predict the occurrence of fractures during follow-up.

All statistical tests were two-sided, with significance set at *p* < 0.05. Statistical analyses were carried out using SPSS version 29.0 for Windows (IBM Corporation, New York, NY, USA).

In the in vitro study, data are presented as mean ± standard deviation (SD) from at least three independent experiments and analysed using a two-tailed Student’s t-test. A p-value of < 0.05 was considered statistically significant. Statistical analyses were conducted using GraphPad Prism version 9.3.1.

## Results

A total of 191 individuals (70% female) were enrolled in the study. Table 1 shows the demographic and clinical characteristics of the study population. The mean age was 63 ± 8 years. The prevalence of the female gender was 70%, and obesity was observed in 34% of participants. Furthermore, 28% individuals exhibited low ASMM/sarcopenia (Supplemental Table 1).

**Table 1:** Multivariable linear regression analysis - Factors associated with ASMM by gender, in adults and older individuals

**Women**						
Dependent variable *ASMM*	B	SE	β	*p-value*	C.I. 95%	
					LL	UL
Age	-0.15	0.02	-0.47	< 0.001	-0.20	-0.10
FM	0.10	0.02	0.30	< 0.001	0.05	0.15
C-peptide	0.57	0.22	0.18	0.013	0.12	1.01
**Men**						
Age	-0.22	0.05	-0.44	< 0.001	-0.33	-0.10
C-peptide	1.16	0.44	0.29	0.011	0.27	2.05

Women― Variables included in the model: age, FM, and serum levels of C peptide and insulin

Men― Variables included in the model: age, FM, and serum levels of C peptide and insulin

*ASMM* appendicular skeletal muscle mass, *FM* fat mass, *B* unstandardized coefficients, *SE* standard errors, *C.I.* confidence interval, *LL* lower limit, *UL* upper limit

Since women are prevalent in our population, we performed a Pearson correlation analysis stratified by gender. Univariate analysis revealed that the factors correlated with ASMM in both genders were age, BMI, FM, and serum concentrations of C-peptide and insulin (all correlations with *p* < 0.1) while no significant correlations were observed for physical activity, medication use, glucose, or creatinine.

Table 1 shows the association between ASMM and the factors identified in Pearson’s correlation, stratified by gender. In women, ASMM remained negatively associated with age, and positively associated with FM and serum concentrations of C-peptide. In men, ASMM was associated only with age and serum levels of C-peptide (Table 1).

The binary logistic regression analysis was performed to assess the association between dependent binary variable “low ASMM/sarcopenia” and C peptide. In Table 2, low serum levels of C-peptide were significantly associated with increased odds of having low ASMM/sarcopenia (B = -1.07, *p* < 0.001; OR = 0.34, 95% CI: 0.18–0.61). We also found that age and FM were significantly associated with low ASMM/sarcopenia (Age: B = 0.11, *p* < 0.001; OR = 1.12, 95% CI: 1.06–1.18; FM: B = -0.07, *p* = 0.005; OR = 0.92, 95% CI 0.87–0.97, respectively).

**Table 2:** Logistic regression analysis – Factors associated with low ASMM/sarcopenia in adults and older individuals

Dependent variable *Low ASMM/sarcopenia*	B	SE	OR	*p*-value	C.I. 95%	
					LL	UL
Age	0.117	0.028	1.124	< 0.001	1.063	1.189
FM	-0.079	0.028	0.924	0.005	0.874	0.977
C-peptide	-1.075	0.299	0.340	< 0.001	0.189	0.612

Variables included in the model: age, gender, FM, and serum levels of insulin and C-peptide

*ASMM* appendicular skeletal muscle mass, *FM* fat mass, *B* unstandardized coefficients, *SE* standard errors, *OR* odds ratio, *C.I.* confidence interval, *LL* lower limit, *UL* upper limit

Thus, in order to assess the probability of having low ASMM/sarcopenia across different serum C-peptide levels, the population was divided into C-peptide tertiles. The characteristics of the population according to C-peptide tertiles are reported in Supplemental Table 2. Participants in the first tertile (lowest C-peptide value) had a higher age (*p* = 0.002), and a lower BMI (*p* < 0.001), FM (*p* < 0.001), glucose (*p* < 0.001), insulin (*p* < 0001), ASMM (*p* < 0.001) and SMM (*p* < 0.001), than those in the third tertile (highest C-peptide value; Supplemental Table 2). The prevalence of gender, obesity, hypertension, use of antihypertensive medications was also different among C-peptide tertiles (Supplemental Table 2). Supplemental Table 3 presents the dietary intake assessment across C-peptide tertiles. No statistically significant differences were found between C-peptide tertiles in terms of energy and nutrient intake (Supplemental Table 3).

**Fig. 1:**
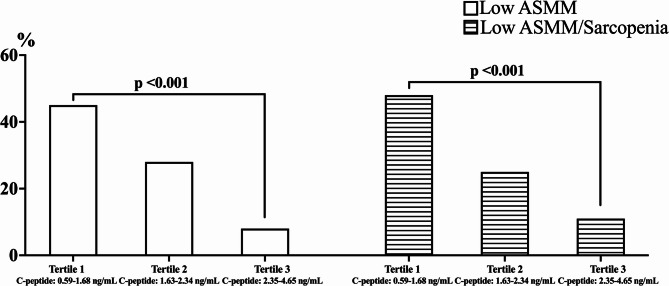
Prevalence of low ASMM and sarcopenia in the population according with tertiles of C-peptide in adults and older individuals. Participants in the lowest C-peptide tertile showed the highest prevalence of low ASMM and sarcopenia, while those in the highest tertile showed the lowest prevalence. Statistical differences across tertiles were evaluated using the Chi-square test. Abbreviation. ASMM, appendicular skeletal muscle mass

Figure 1 showed the prevalence of low ASMM and low ASMM/sarcopenia across C-peptide tertiles. A significant difference in the prevalence of low ASMM was observed, with the highest prevalence in the first tertile compared to the others (*p* < 0.001). Specifically, 45% of participants in the first tertile had low ASMM, compared to 24% in the second and 8% in the third tertile. Similarly, a significant difference in the prevalence of low ASMM/sarcopenia was found among the tertiles. The prevalence of low ASMM/sarcopenia was 48% in the first tertile, 25% in the second, and 11% in the third (*p* < 0.001). After adjusting for age, gender, FM and serum levels of glucose and insulin, the difference in the prevalence of low ASMM and low ASMM/sarcopenia between C-peptide groups remained significant (*p* = 0.027, and *p* = 0.029; respectively).

**Fig. 2:**
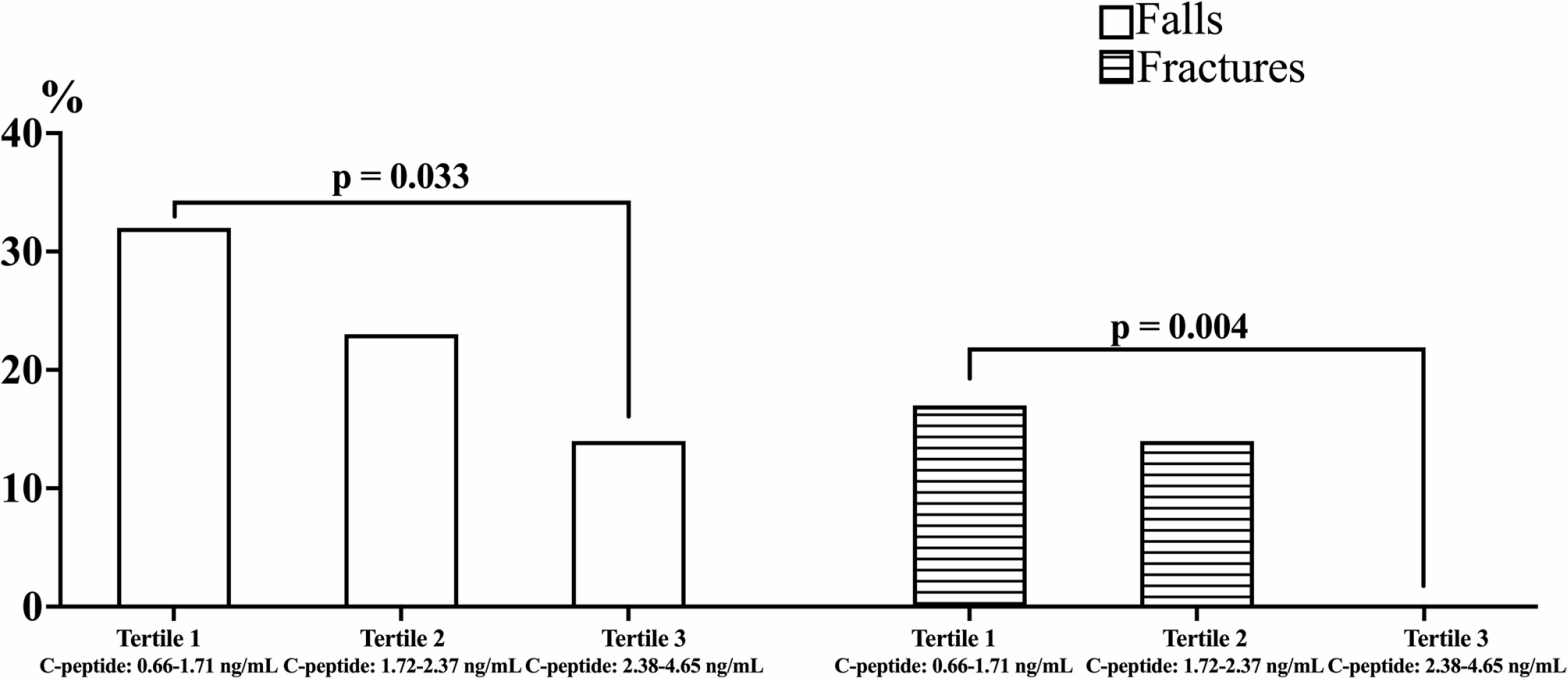
The area under the ROC curve for C-peptide to predict low ASMM/sarcopenia in adults and older individuals. The ROC analysis showed that lower serum C-peptide levels are associated with reduced muscle mass and sarcopenia. Abbreviation: ASMM, appendicular skeletal muscle mass

The area under the ROC curve for C-peptide in predicting the presence of low ASMM/sarcopenia was 0.248 (SE = 0.038; *p* < 0.001; lower limit 0.17, upper limit 0.32, Fig. 2). A serum C-peptide level of less than 2.84 ng/mL demonstrated good specificity (75%) for predicting low ASMM/sarcopenia but low sensitivity (4%). In contrast, a C-peptide level of 0.98 ng/mL exhibited excellent sensitivity (85%) while maintaining low specificity (4%) for predicting low ASMM/sarcopenia.

### Falls and fractures at follow-up

**Fig. 3:**
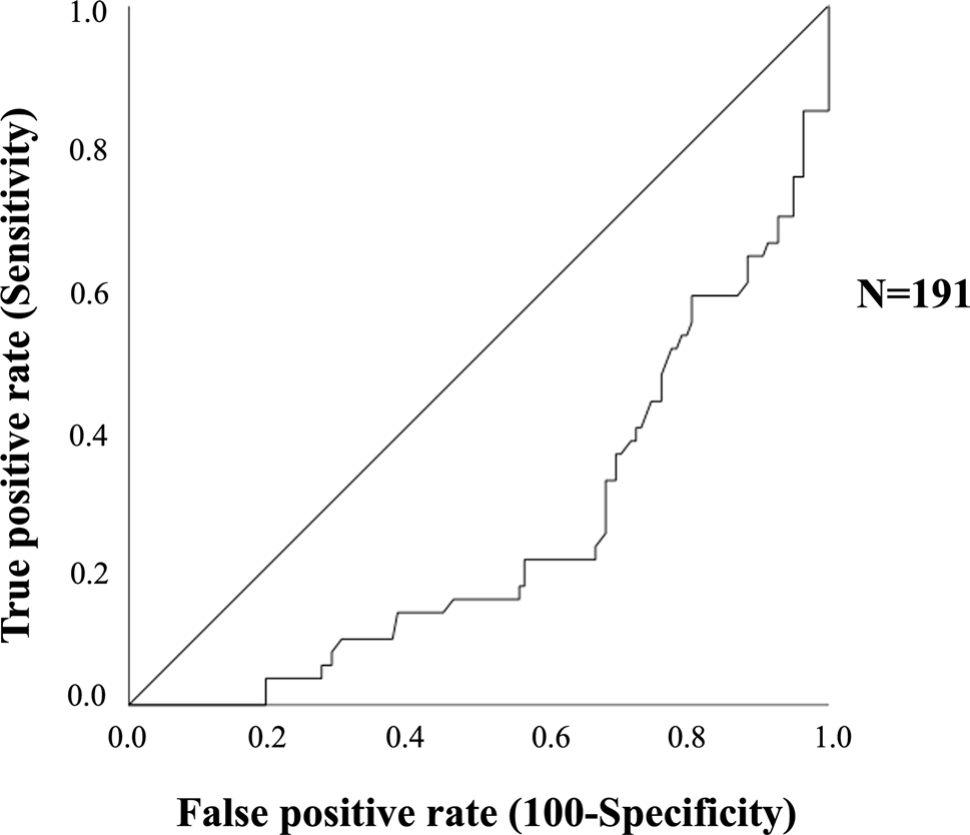
Prevalence of falls and fractures in the population according with tertiles of C-peptide in adults and older individuals. Participants in the lowest C-peptide tertile showed the highest prevalence of both falls and fractures, whereas no fractures occurred in the highest tertile. Statistical differences across tertiles were assessed using the chi-square test

Data on falls and fractures were available for 164 adults and elderly individuals at follow-up, who were again classified into tertiles of serum C-peptide. Figure 3 shows the prevalence of falls and fractures during follow-up across C-peptide tertiles. After a median follow-up of 28 ± 38 months patients in the first tertile exhibited a significant higher prevalence of falls (32%) compared to the second (23%) and the third tertile of C-peptide (14%) (*p* = 0.033; Fig. 3). At the follow-up, a total of 17 individuals (10%) had fractures, including femoral (*n* = 5), vertebral (*n* = 5), wrist (*n* = 2), and other sites (*n* = 5). Similarly, patients in the first tertile exhibited a significant higher prevalence of fractures (17%) compared to the second (14%) and third tertile (none in this group) (*p* = 0.004; Fig. 3). Since we observed a significant difference in C-peptide levels between men and women, and only one fracture occurred in men, we analysed these data (i.e. prevalence of low ASMM/sarcopenia, falls and fractures) exclusively in females, and the results remained unchanged (data not shown).

Finally, we evaluated the factors associated to falls and fractures. Logistic regression analysis showed that falls in adults and elderly participants were significantly associated with ASMM (B = -0.18; *p* = 0.002) (Table 3). Conversely, fractures were significantly associated with falls (B = 1.45; *p* = 0.009) and serum levels of C-peptide (B = -1.22; *p* = 0.010) (Table 3). ROC analysis showed that a serum C-peptide level > 2.20 ng/mL effectively excluded the occurrence of fractures during follow-up (AUC = 0.747; SE = 0.049; *p* < 0.001; lower limit 0.65, upper limit 0.84; specificity = 100%, sensitivity = 45%).

**Table 3:** Logistic regression analysis – Factors associated with falls and fractures in adults and older individuals

Dependent variable ^*^ *Falls*	B	SE	OR	*p*-value	C.I. 95%	
					LL	UL
ASMM	-0.185	0.060	0.831	0.002	0.738	0.935
Dependent variable ^*§*^ *Fractures*	B	SE	OR	*p-value*	C.I. 95%	
					LL	UL
Falls	1.453	0.557	4.276	0.009	1.435	12.742
C-peptide	-1.227	0.474	0.293	0.010	0.116	0.742

^*^ Variables included in the model: gender, and serum levels of glucose, insulin and C-peptide

^*§*^ Variables included in the model: gender, and serum levels of glucose, insulin and C-peptide

*ASMM* appendicular skeletal muscle mass, *B* unstandardized coefficients, *SE* standard errors, *OR* odds ratio, *C.I.* confidence interval, *LL* lower limit, *UL* upper limit

### HPLC analysis suggests cellular utilization of C-peptide in C2C12 cells

To assess whether C-peptide is utilized by the cells, its presence in the culture medium was analyzed by HPLC before and after cell treatment. After 48 h of incubation, a marked reduction in extracellular C-peptide concentration was observed, decreasing from approximately 10 nM to 1.74 ± 0.27 nM (*p* < 0.001), indicating that the peptide is utilized and sequestered by the cellular system (Supplemental Fig. 1).

### C-peptide promotes the time of myogenic differentiation in C2C12 cells

**Fig. 4:**
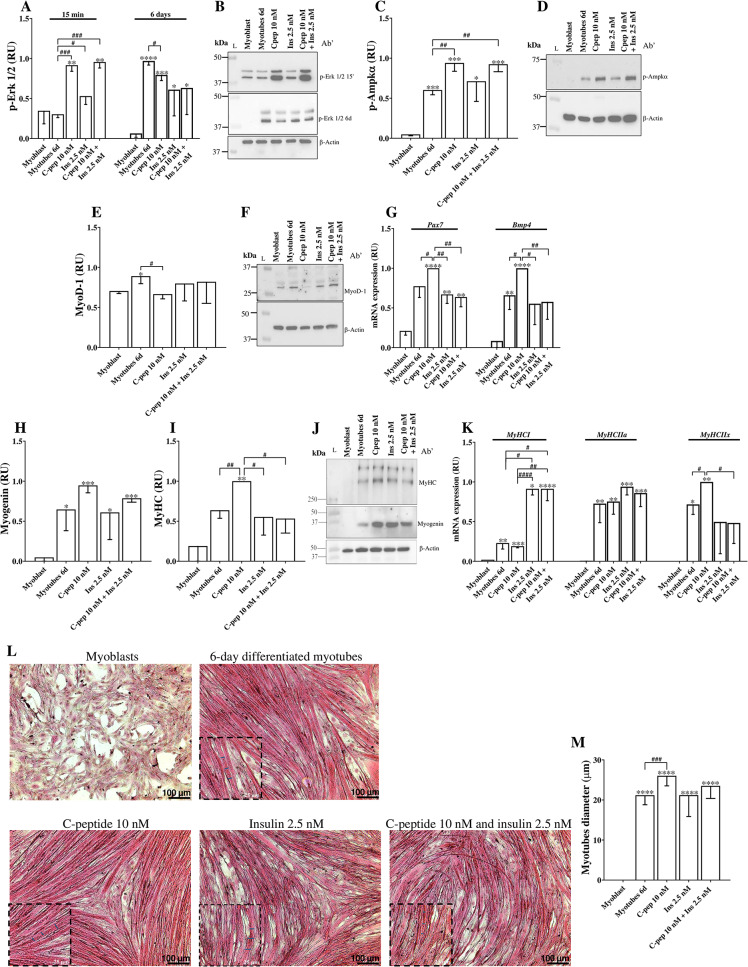
C-peptide promotes myogenic differentiation in C2C12 cells **(A**,** B)** quantification of myotubes diameter. **(C**,** D)** Protein levels of Myogenin and MyHC, markers of early and later differentiation to myotube. **(C-E)** Quantification of protein levels relative to β-Actin. Protein levels were measured by western blotting (see methods for antibodies). **(F)** mRNA expression levels of *MyHC I*, *MyHC IIa*, *MyHC IIx*,* Pax7* and *Bmp4*, were measured using Real-Time PCR. Data were analyzed using the 2^^−ΔΔCt^ method and normalized to β-Actin. Data are represented as mean ± SD of three independent experiments. *p*-values calculated by Student’s t-test vs. myoblasts: *p < 0.05, **p < 0.01; ***p < 0.001; ****p < 0.0001. Abbreviations: RU= Relative unit; MyHC= Myosin Heavy Chain; *MyHC I = Myosin Heavy Chain type I; MyHC IIa= Myosin Heavy Chain type IIa; MyHC IIx= Myosin Heavy Chain type IIx; Pax7 = Paired Box Protein 7 and Bmp4 = Bone Morphogenetic Protein 4*

To evaluate whether C-peptide and insulin treatment could accelerate myogenic differentiation times, C2C12 myoblasts were treated with 10 nM of C-peptide and 2.5 nM of insulin alone or in combination for six days (Fig. 4). First, early and late signaling pathways were analyzed. As shown in Fig. 4A, B, treatment with C-peptide or insulin, alone or in combination, induced a rapid increase in ERK1/2 phosphorylation at 15 min compared with untreated myoblasts and 6-day differentiated controls (*p* < 0.001, *p* < 0.05, and *p* < 0.001, respectively). At the 6-day differentiation endpoint, ERK1/2 phosphorylation was significantly reduced in C-peptide–treated cells compared with the 6-day differentiation control (*p* < 0.05). In parallel, phosphorylation of AMPKα was significantly increased at day 6 in cells treated with C-peptide alone or in combination with insulin compared with both myoblasts and 6-day differentiated controls (*p* < 0.001 and *p* < 0.001, respectively; Fig. 4C–D). Analysis of myogenic regulatory factors showed that MyoD1 protein levels were significantly reduced in C-peptide–treated cells compared to the 6-day differentiation control (*p* < 0.01; Fig. 4E, F). In contrast, gene expression analysis revealed that Pax7 mRNA levels were significantly higher in C-peptide–treated cells compared to the 6-day control, insulin alone, and combined treatment (*p* < 0.05, *p* < 0.01, and *p* < 0.01, respectively; Fig. 4G). Similarly, BMP4 expression was significantly upregulated by C-peptide relative to all other conditions (Fig. 4G). Moreover, late-stage differentiation marker were then evaluated. Specifically, MyHC protein levels were significantly increased in C-peptide-treated cells (*p* < 0.01; Fig. 4D), indicating a more advanced stage of myogenic maturation. Interestingly, gene expression analysis of myosin heavy chain isoforms, which play a key role in determining muscle fiber phenotype, revealed distinct effects of the treatments. Insulin, both alone and in combination with C-peptide, significantly upregulated the expression of the *MyHC I* isoform compared to the 6-day control (*p* < 0.05; Fig. 4F), suggesting a shift toward a slow-twitch, oxidative muscle fiber profile. Interestingly, C-peptide treatment selectively increased the expression of the *MyHC IIx* isoform (*p* < 0.05; Fig. 4F), typically associated with fast-twitch, glycolytic muscle fibers.

Finally, morphological analysis confirmed these molecular findings. Myotube diameter was significantly increased in C-peptide–treated cells compared to the 6-day differentiation control (*p* < 0.01; Fig. 4L–M). No significant differences were observed between insulin-treated or combined treatment groups and the 6-day control.

Together, these results indicate that C-peptide modulates early and late signaling pathways, enhances myogenic maturation, and promotes the formation of larger, more differentiated myotubes in C2C12 cells.

#### C-peptide mitigates nutrient deprivation and glucocorticoid-induced muscle damage in C2C12 myotubes

**Fig. 5:**
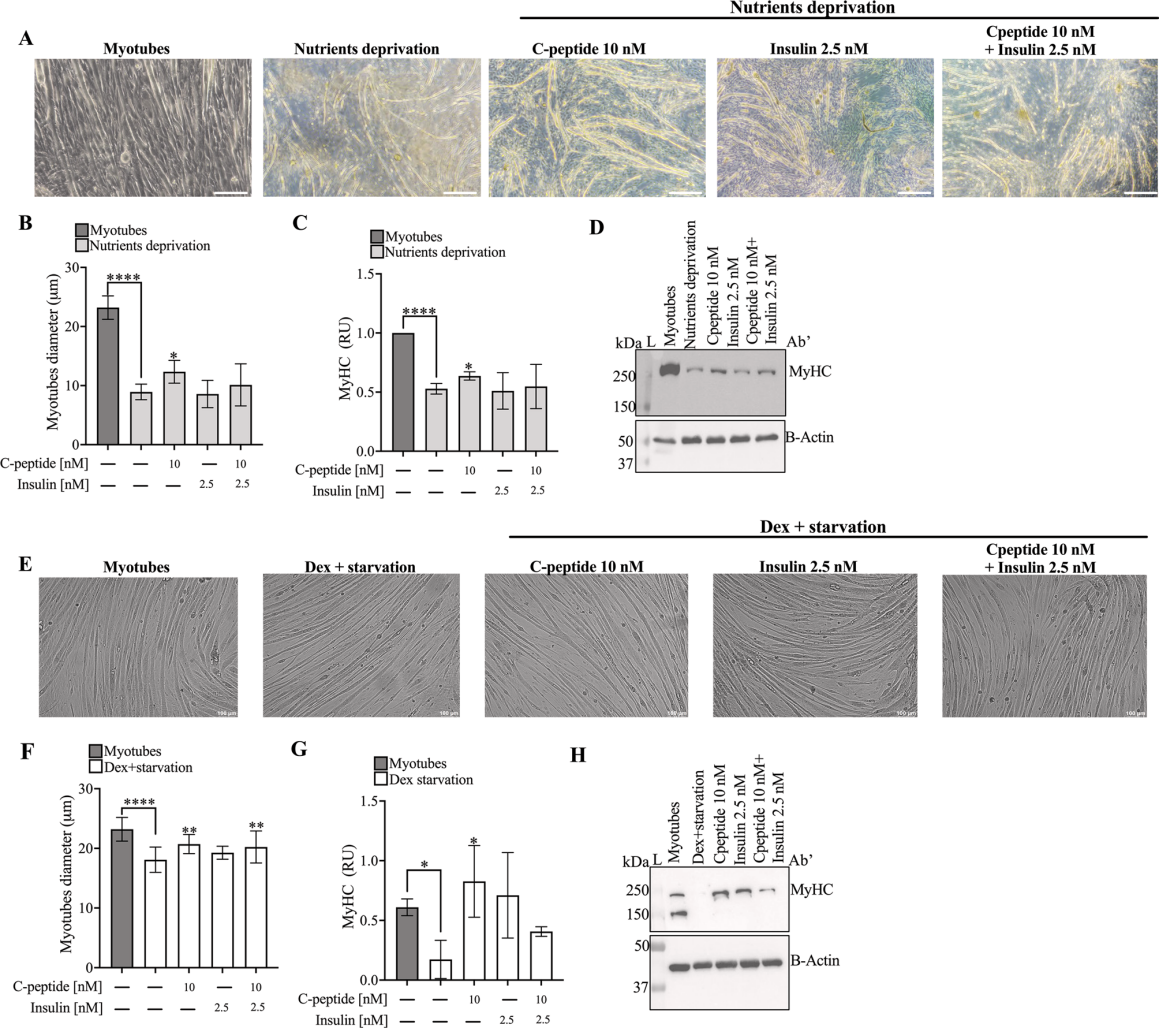
C-peptide mitigates nutrient deprivation– and glucocorticoid-induced muscle damage in C2C12 myotubes. **(A**,** E)** Representative images of C2C12 myotubes (20× magnification) following nutrient-deprivation or glucocorticoid-induced damage and subsequent treatment with C-peptide or insulin. **(B**,** F)** Quantification of myotube diameter under the indicated conditions. **(C**,** G)** Protein expression levels of MyHC, a marker of late myogenic differentiation. Quantification shows protein levels normalized to β-Actin. (**D**,** H)** Protein expression was assessed by western blotting. Data represent mean ± SD from three independent experiments. Statistical significance was calculated using Student’s t-test: *p < 0.05, **p < 0.01, ****p < 0.0001. Abbreviations: RU = Relative Units; MyHC= Myosin Heavy Chain

To evaluate whether C-peptide protects mature myotubes from atrophic stimuli, post-mitotic C2C12 cells were exposed to either nutrient deprivation or glucocorticoid excess (Fig. 5).

Under nutrient-deprivation conditions, PBS treatment markedly reduced myotube diameter (*p* < 0.0001) and significantly decreased MyHC protein levels (*p* < 0.001) compared to untreated myotubes (Fig. 5A–D). Treatment with C-peptide prevented the reduction in MyHC expression (*p* < 0.05) and partially preserved myotube morphology, whereas insulin, either alone or combined with C-peptide, did not counteract PBS-induced damage.

Next, in the glucocorticoid-induced atrophy model, DEX combined with starvation led to a pronounced decrease in myotube diameter (*p* < 0.0001) and a significant reduction in MyHC protein expression (Fig. 5E–H). C-peptide attenuated MyHC loss (*p* < 0.05) and improved myotube structural integrity. Insulin alone did not exert protective effects; however, the combination of C-peptide and insulin partially mitigated DEX-induced damage, indicating a synergistic or additive interaction under glucocorticoid stress.

## Discussion

This study demonstrates, for the first time, that C-peptide, long considered biologically inactive, is involved in the pathophysiological mechanisms of sarcopenia and fractures, confirming our hypothesis that its circulating levels are associated with sarcopenia, an increased risk of fractures, and that the C-peptide directly promotes myogenic differentiation while attenuating atrophic damage. In detail, we find an association between the serum concentrations of C-peptide and ASMM and low ASMM/sarcopenia. Furthermore, we observed a statistically significant difference in ASMM as well as the prevalence of low ASMM/sarcopenia based on serum C-peptide levels in a population of adults and elderly. Specifically, individuals with lower C-peptide levels showed a higher prevalence of low ASMM/sarcopenia compared to those with higher serum C-peptide levels. Notably, these differences were independent of physical activity, protein intake, or caloric intake among the C-peptide tertiles.

Sarcopenia is indeed a multifactorial clinical condition [1]. Among its primary causes are muscle aging, protein-energy malnutrition, and physical inactivity/reduced mobility. Therefore, establishing an independent association between C-peptide and muscle dysfunction requires accounting for potential confounding factors, which we have done in this study.

Moreover, we demonstrated for the first time, through in vitro studies on C2C12 cells, that C-peptide exerts protective effects on muscle cells not only in a malnutrition model but also under glucocorticoid-induced atrophic conditions. In both models, C-peptide prevented the reduction in myotube diameter and enhanced myosin expression, confirming its ability to preserve muscle fiber integrity. These findings are consistent with previous evidence showing that C-peptide counteracts dexamethasone-induced atrophy. In C2C12 cells treated with DEX, C-peptide restored MHC levels and significantly improved both the differentiation and fusion indices [28]. In vivo, the same study also reported that co-administration of C-peptide with DEX mitigated muscle mass loss, improved grip strength, and increased the cross-sectional area of gastrocnemius fibers, further supporting its protective role against glucocorticoid-induced muscle wasting. This research is original as we also demonstrated that C-peptide promotes the differentiation of myoblasts into mature myotubes compared to untreated cells. Our data indicate that C-peptide modulates both early and late intracellular signaling pathways during myogenic differentiation, characterized by a rapid activation of pErk1/2 followed by a reduction in pErk1/2 and an increase in pAMPK phosphorylation at the 6-day differentiation. Rapid phosphorilation of Erk1/2 is consistent with previous reports describing receptor-mediated, GPCR-like signaling induced by C-peptide [10, 29], whereas attenuation of pErk signaling at later stages has been associated with the transition from proliferative to differentiation-permissive programs in myogenic cells [30].

Concurrently, C-peptide modulated key molecular regulators of the myogenic niche, namely *BMP4* and *Pax7* [31, 32]. In particular, C-peptide increased *BMP4* expression, and previous studies indicate that pErk1/2 and BMP4 are part of the same regulatory network, in which reduced pErk1/2 signaling may favor BMP4 expression through feedback mechanisms [33–35]. Accordingly, the reduced pErk1/2 pathway together with increased *BMP4* expression observed after C-peptide treatment could reflect a coordinated remodeling of myogenic niche signaling rather than a direct Erk-dependent effect.

In addition, C-peptide treatment increased *Pax7* expression, and this effect was accompanied by enhanced AMPKα phosphorylation. pAMPK has been identified as a key regulator of satellite cell identity and myogenic fate, with loss of pAMPK resulting in reduced Pax7 expression and impaired myogenic programs [36]. Moreover, AMPKα is the predominant AMPK isoform in satellite cells, and its deficiency compromises satellite cell activation and muscle regeneration in vivo [37]. Consistently, C-peptide treatment reducing MyoD1 protein levels. In this context, the concomitant reduction of MyoD1 at the differentiation endpoint is consistent with a shift toward a Pax7⁺/MyoD⁻ reserve-like state, reflecting a balance between the maintenance of progenitor identity and the maturation of committed myotubes [38]. Notably, C-peptide treatment upregulated the expression of the *MyHC IIx* isoform, which is characteristic of fast-twitch, glycolytic fibers. These fibers are known to be preferentially affected in age-related sarcopenia with a shift toward slow-twitch fiber composition [31, 39].

Thus, C-peptide appears to be associated with anabolic-related processes, with notable regenerative potential, which could represent a future therapy for sarcopenia. This is further supported by the increased diameter of myotubes developed from myoblasts treated with C-peptide compared to those exposed only to the culture medium.

The baseline serum C-peptide level serves as a predictor not only for basal low ASMM/sarcopenia but also for the occurrence of fractures during follow-up. In particular, we found that for each unit increase in serum C-peptide, the odds of having low ASMM/sarcopenia decrease by approximately 66%. Similarly, for fractures, each unit increase in C-peptide was associated with a 71% reduction in the odds of experiencing a fracture. In addition, a serum C-peptide level more than 2.20 ng/mL effectively excludes the risk of fractures over a 28-month follow-up period. These results strongly support a protective role of serum C-peptide in musculoskeletal heath.

Currently, no pharmacological therapy exists for treating muscle mass loss. It is well known that proper nutrition, protein and vitamin D supplementation, as well as physical exercise, help maintain muscle strength and mass [40] However, studies on the effects of nutrients and physical activity in individuals at risk of sarcopenia do not point in a single clear direction, making it difficult to draw definitive conclusions. Protein supplementation improves muscle mass in community-dwelling older adult [41]. Furthermore, protein supplementation combined with physical exercise exerts a significant positive effect on muscle mass, muscle strength and physical mobility in elderly people at high risks of sarcopenia and frailty [42]. However, it has been demonstrated that supplementation with essential amino acids and protein without rehabilitation exercise yields a minor impact on muscle health compared to a combined treatment [43]. Furthermore, studies have shown short-term resistance training alone increases strength but not muscle size [44]. Combining protein supplementation, vitamin D, and resistance training is particularly effective in increasing muscle mass in elderly individuals with sarcopenia, but this synergistic effect is not observed in those with normal muscle mass [45]. This suggests that such interventions may be most beneficial for those already experiencing muscle decline.

Physical activity is often impractical for the elderly, especially for those who are disabled or socially isolated. Protein supplementation must be approached with caution in individuals affected by kidney diseases. Serum levels of vitamin D fluctuate, particularly between seasons and depending on the type of supplementation. A pharmacological therapy based on peptide C could therefore represent an important therapeutic option, offering better compliance compared to all the therapeutic possibilities mentioned above. Since treatment with C-peptide increases myotube diameter, this finding raises hope for its potential therapeutic use in maintaining or increasing muscle mass, as supported by our observational study associating serum C-peptide with ASMM. Clinical trials would be required to definitively confirm this hypothesis. Despite these promising findings, the translational application of C-peptide as a therapeutic agent requires consideration of its pharmacokinetic properties. Like many therapeutic peptides, C-peptide displays limited in vivo stability and a short plasma half-life (approximately 20–40 min), primarily due to proteolytic degradation and renal clearance [46, 47]. To overcome these limitations, several formulation and delivery approaches have been explored in peptide therapeutics, including structural modifications such as PEGylation or cyclization, conjugation to larger carriers (e.g. lipids and polymers), N-terminal modifications and encapsulation within nanoscale delivery systems such as polymeric nanoparticles or liposomes [48]. Although these strategies require further optimization for C-peptide, they represent promising avenues to enhance its translational potential without compromising biological activity.

Regarding the mechanisms of action of C-peptide that result in better muscle mass, it is known to have angiogenic effects, improve endothelial function, and enhance capillary circulation at the renal, cutaneous, and gonadal levels [49]. This could translate into preserved ASMM at the muscular level. In a previous study on rats, in which osteosarcopenia was induced by diabetes, continuous administration of C-peptide at physiological concentrations led to greater muscle mass in the lower limbs compared to diabetic rats with C-peptide deficiency. In that study, we demonstrated that C-peptide administration reduced TRAF6 expression [50]. TRAF6, by modulating vascular endothelial growth factor (VEGF), reduces endothelial cell proliferation and migration, while its inhibition by C-peptide leads to enhanced muscle vascularization [50]. We cannot exclude that C-peptide may exert pro-myogenic effects on muscle tissue, as it appears to stimulates intracellular protein synthesis, but targeted studies are needed to confirm this action.

In the present study, we do not intend to propose C-peptide as a diagnostic test for sarcopenia, nor as an independent diagnostic marker. Rather, our objective is to draw attention to the observed association between lower circulating levels of this peptide and an increased risk of sarcopenia in non-diabetic individuals, and to highlight its potential therapeutic relevance. Evidence from previous experimental study in animal models [11], together with future mechanistic and clinical investigations, may help determine whether C-peptide could represent a candidate for targeted replacement or pharmacological strategies aimed at the treatment of sarcopenia.

Studies on the relationship between serum C-peptide and fractures are scarce. While research has focused on C-peptide’s role in glucose metabolism and its connection to muscle mass, its direct link to fractures remains underexplored. Most studies in this area are preliminary [51, 52]. This study, thus, presents truly unprecedented findings, as we demonstrate that the serum value of C-peptide is a predictor of fractures. These results have direct implications for the diagnosis of osteoporosis, suggesting that C-peptide could serve as a simpler and more cost-effective predictor of fractures compared to bone mineral density (BMD) assessment. Moreover, C-peptide may not only play a role in regulating muscle mass and, inturn, bone mass, but could be directly involved in the pathogenesis of fractures. In a preclinical study of ours, we showed that administering C-peptide to rats with diabetes-induced bone mass loss prevents damage to bone microarchitecture by preserving trabecular thickness [11].

While there are many effective treatments available, these therapies are known to have potential adverse effects. Some may, paradoxically, increase the risk of fractures over time, while others raise the risk of osteonecrosis of the jaw or cause gastrointestinal disturbances [53]. Additionally, long-term adherence to osteoporosis medications remains a challenge. Therefore, these findings offer promising prospects for the development of future therapeutic options.

In our study, the effect of C-peptide on myoblast differentiation was greater than that of insulin.

This finding aligns with other studies suggesting that insulin transport may also occur through fluid-phase uptake into and out of microvascular endothelial cells, potentially involving basolateral exocytosis via mechanisms that remain poorly understood [54]. Moreover, insulin may fail to promote cell differentiation due to a significant reduction in IR-beta levels, which is induced by insulin itself [55].

A limitation of this study is its observational nature, although it serves to generate hypotheses for potential future human trials. In addition, it is well known that cellular studies do not perfectly reflect what occurs in vivo. The low prevalence of sarcopenia can be attributed to the age range of the enrolled population. Another limitation is the lack of standardized measures of physical performance (e.g., gait speed, chair stand test, or short physical performance battery), which precludes conclusions regarding the association between serum C-peptide levels and functional outcomes. Unfortunately, we do not have data on serum vitamin D levels in the study population, but the deficiency alone is not a cause of sarcopenia. However, a strength of this study lies in the large sample size and the predominance of female participants. To our knowledge, many human studies evaluating the effects of peptides on muscle have mainly enrolled male subjects. Therefore, our findings help address this gap by providing evidence in an underrepresented female population. Furthermore, previous experimental and clinical studies have primarily examined the role of C-peptide in muscle loss in the context of diabetes or insulin deficiency. In contrast, our study expands current knowledge by demonstrating, for the first time, an independent association between circulating C-peptide levels and sarcopenia, as well as the incidence of falls and fractures, in a non-diabetic adult and elderly population. Moreover, the in vitroresults reinforce the observational findings in humans by identifying coordinated ERK1/2 and AMPK signaling, together with modulation of *Pax7* and *BMP4*, as potential mechanisms underlying its pro-myogenic and regenerative effects. Taking into account dietary intake and physical activity is another strength, although we cannot entirely rule out other confounding factors.

In conclusion, this study highlights the protective role of C-peptide in musculoskeletal health, showing that higher levels are associated with a reduced risk of low ASMM/sarcopenia and fractures adults and the elderly. In a cellular model, C-peptide promotes muscle differentiation and increases myotube diameter, suggesting a potential supportive role in muscle anabolism and highlighting its possible relevance as a therapeutic candidate for sarcopenia, especially in populations where current therapeutic strategies are limited. Further clinical trials are required to confirm these results and evaluate the potential of C-peptide as novel treatment for the prevention of sarcopenia and fractures.

## Supplementary Information

Below is the link to the electronic supplementary material.

Supplementary Material 1

Supplementary Material 2

Supplementary Material 3

Supplementary Material 4

Supplementary Material 5

## Data Availability

The data are available from the authors upon request.

## Abbreviations

ASMM: Appendicular skeletal muscle mass
BMI: Body mass index
Bmp4: Bone Morphogenetic Protein 4
CREA: Research Centre for Food and Nutrition
DEX: Dexamethasone
DMEM: Dulbecco’s Modified Eagle Medium High Glucose
EWGSOP2: European Working Group on Sarcopenia in Older People
FBS: Fetal Bovine Serum
FM: Fat mass
FFQ: Food frequency questionnaire
H&E: Hematoxylin and eosin
HGS: Handgrip strength
HS: Horse serum
HS-CRP: High sensitive – C reactive protein
IEO: European Institute of Oncology
MVPA: Moderate to vigorous physical activity
MyHCI: Myosin heavy chain type I
MyHCIIa: Myosin heavy chain type IIa
MyHCIIx: Myosin heavy chain type IIx
Pax-7: Paired Box 7
PBS: Phosphate-buffered saline
P/S: Penicillin-streptomycin
SD: Standard deviation
SMM: Skeletal muscle mass
TC: Total cholesterol
