# Supplementary material for: C-peptide promotes myogenic differentiation in vitro and low serum levels are associated with sarcopenia in adults and the elderly
Source: J Transl Med. 2026 Mar 11;24:542. doi: 10.1186/s12967-026-07983-9 (PMC13094129; doi:10.1186/s12967-026-07983-9)

Figure 4B p-Erk1/2 (15’ min) 42-44 kDa

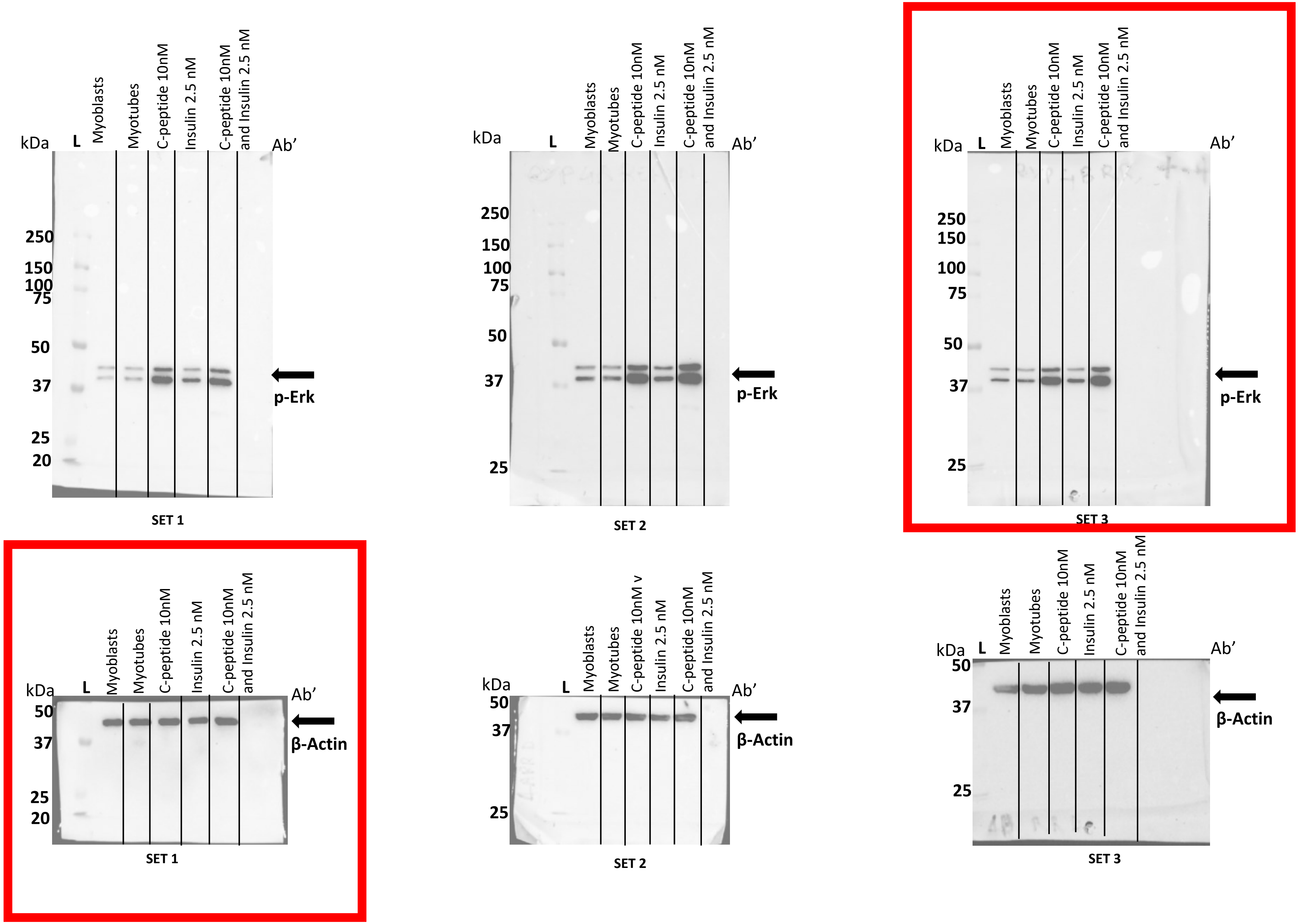

Figure 4B p-Erk1/2 (6 days) 42-44 kDa

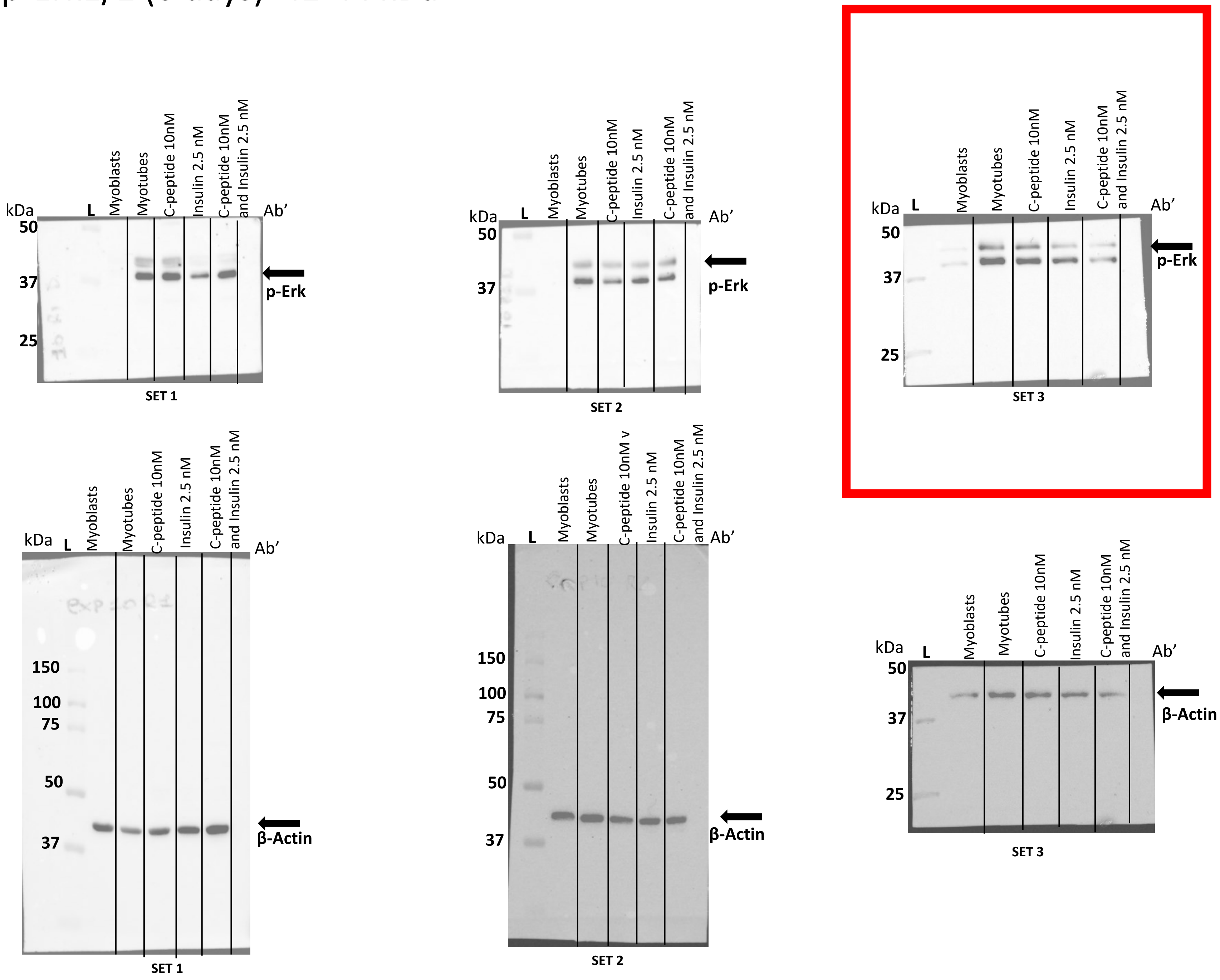

Figure 4D p-Ampkα 62kDa

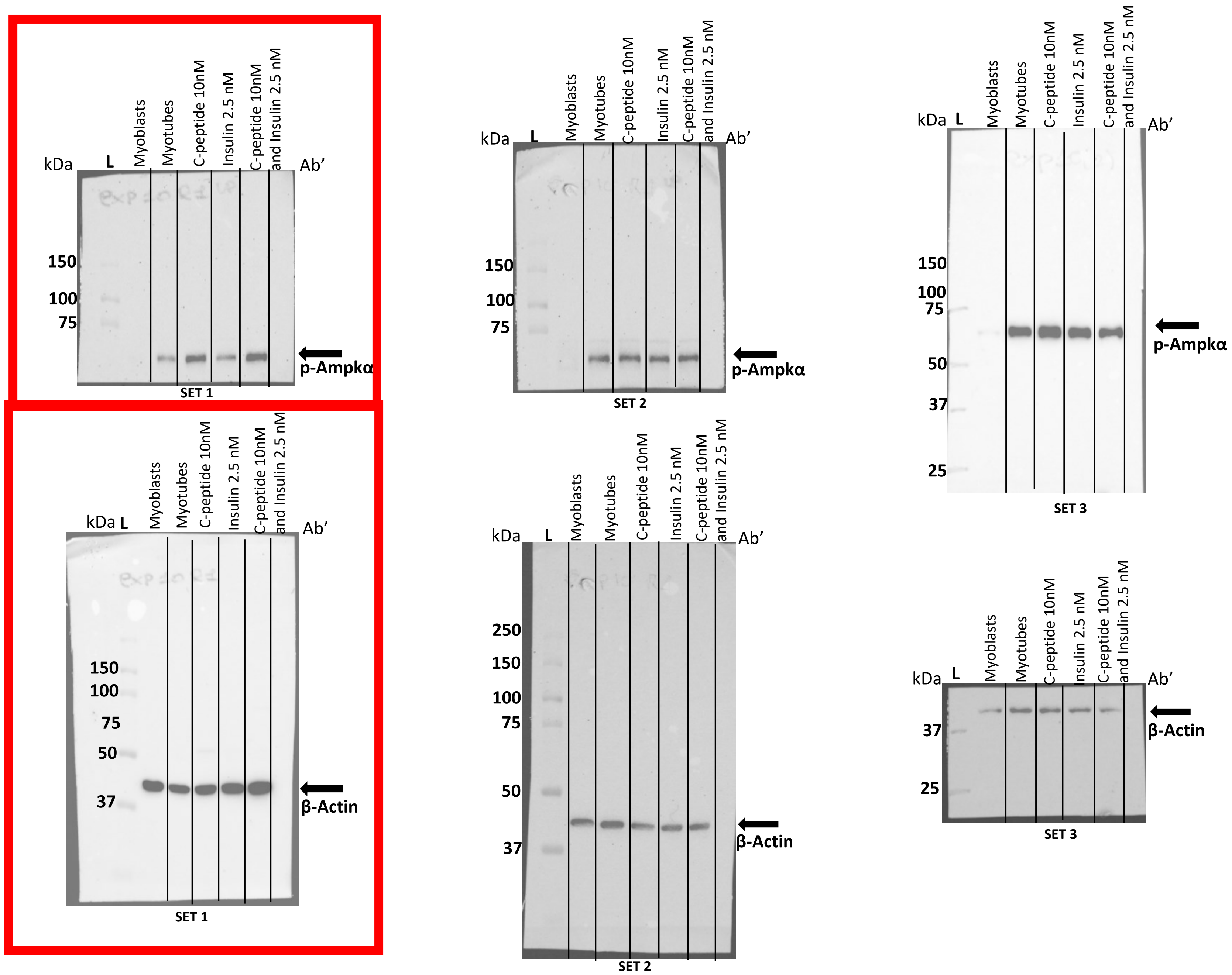

Figure 4F MyoD-1 34.4 /45 kDa

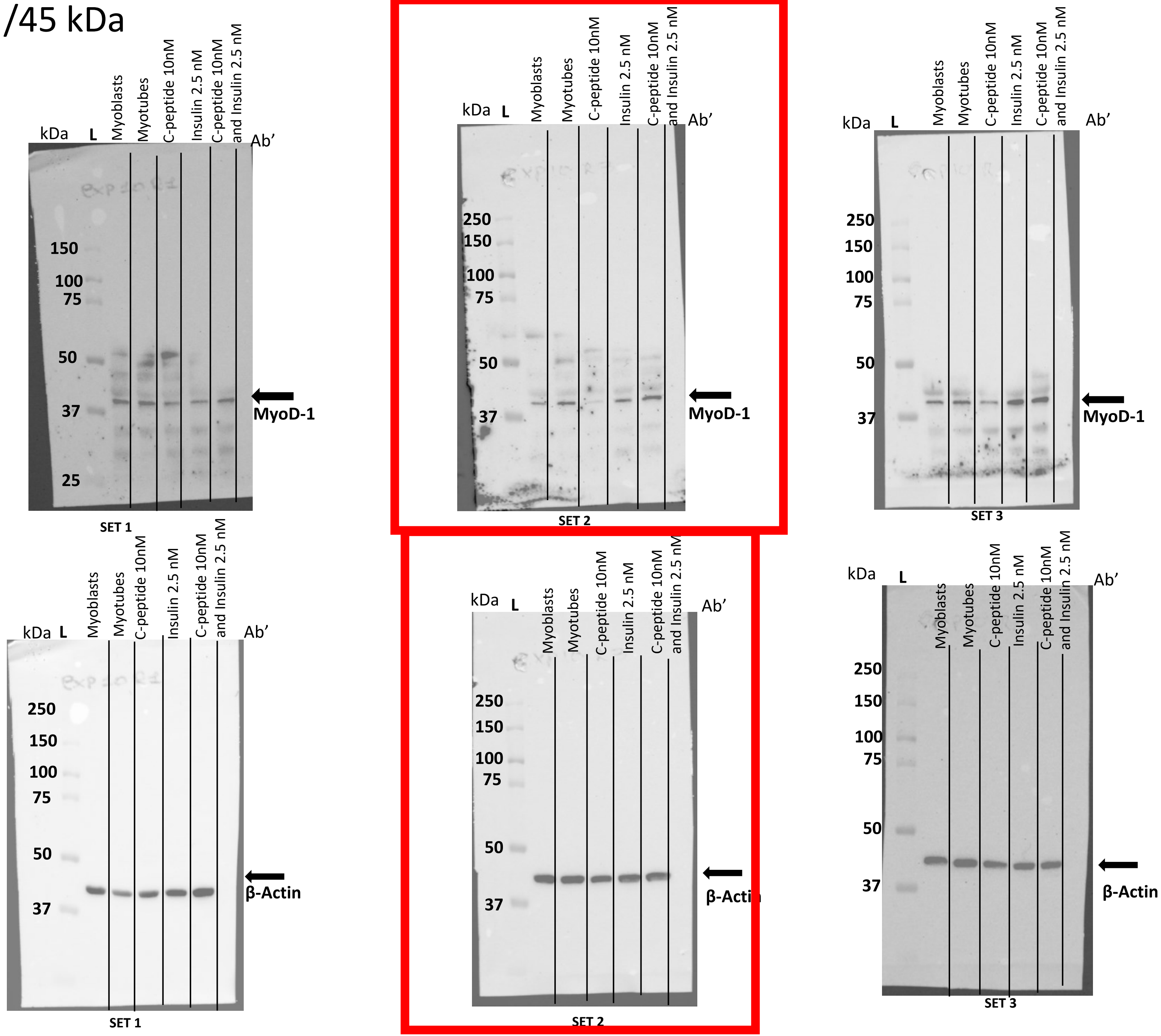

Figure 4J MyHC 250 kDa

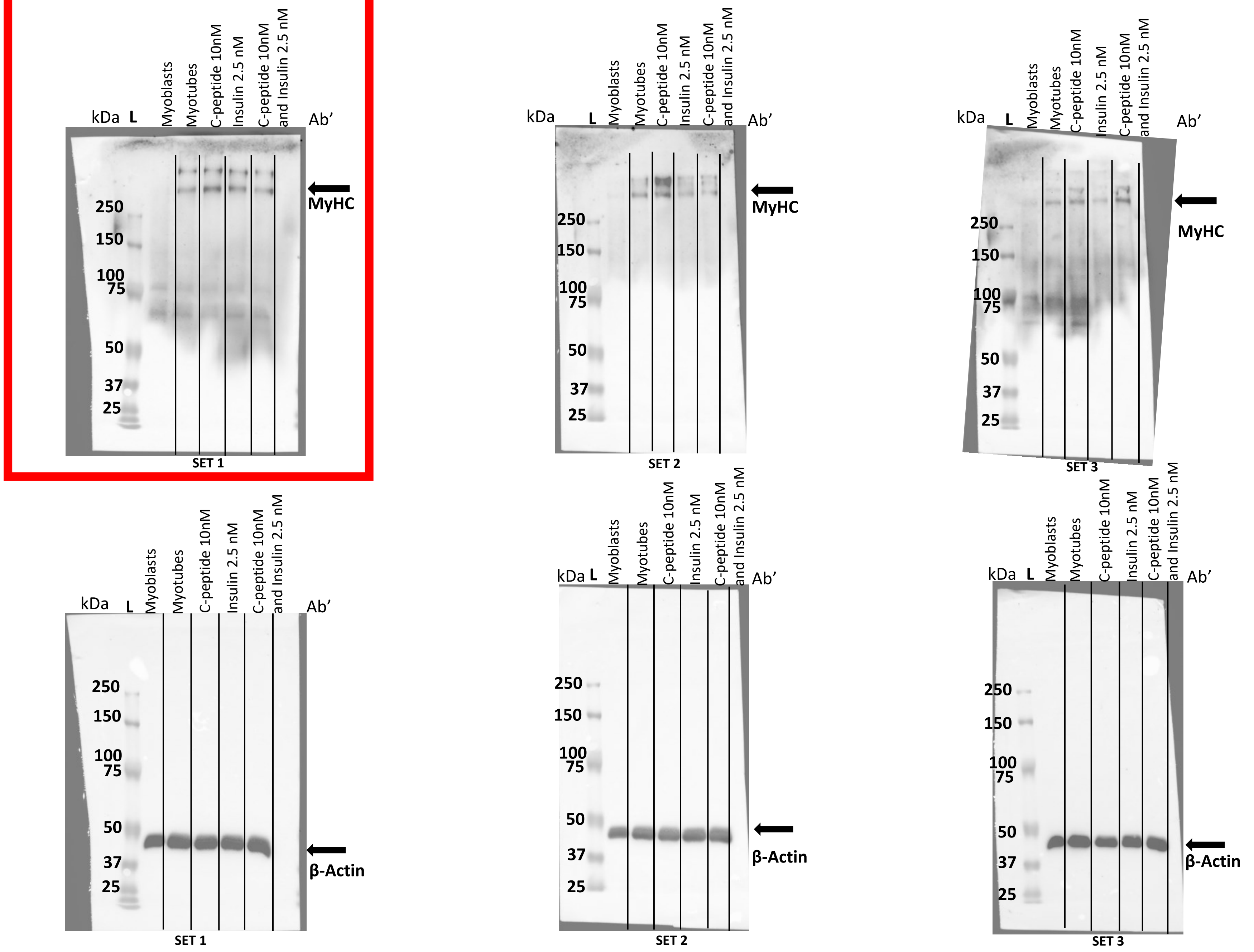

Figure 4J Myogenin 48 kDa

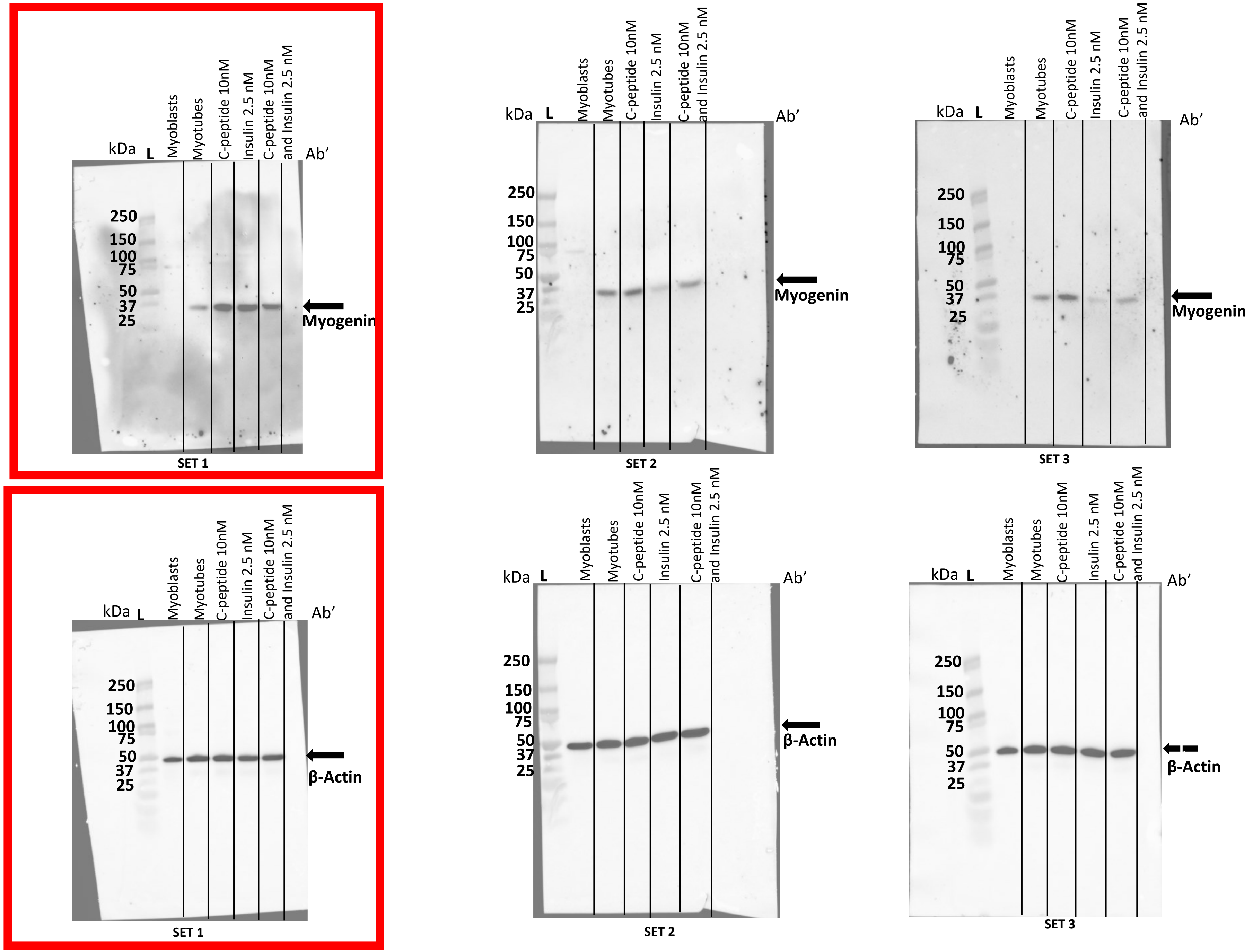

Supplement: Supplementary file 1 — Supplementary Material 1 [file 12967_2026_7983_MOESM1_ESM.pdf]
