# Supplementary figures and images for: C-peptide promotes myogenic differentiation in vitro and low serum levels are associated with sarcopenia in adults and the elderly
Source: J Transl Med. 2026 Mar 11;24:542. doi: 10.1186/s12967-026-07983-9 (PMC13094129; doi:10.1186/s12967-026-07983-9)

Figure 5D MyHC 250 kDa

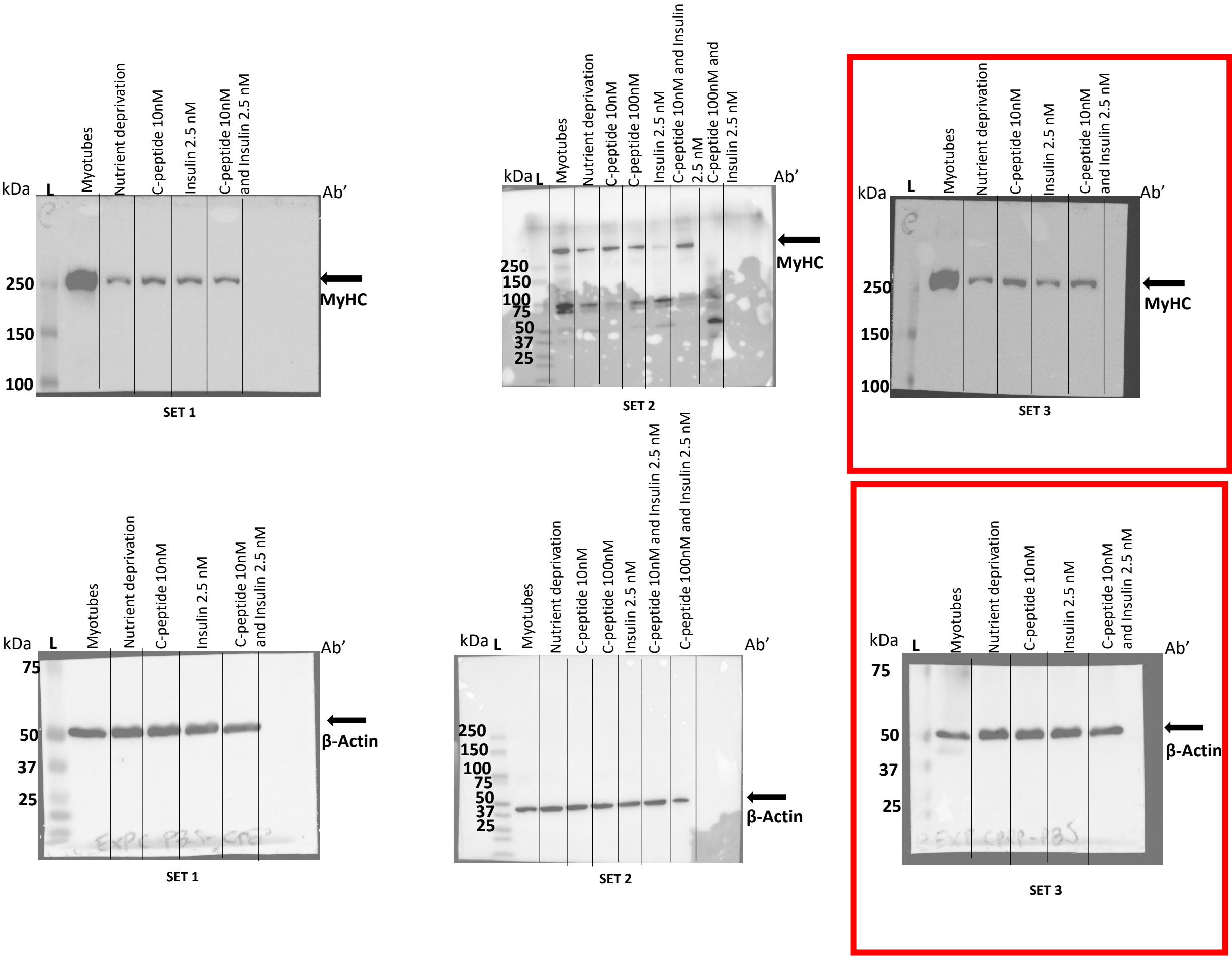

Figure 5H MyHC 250 kDa

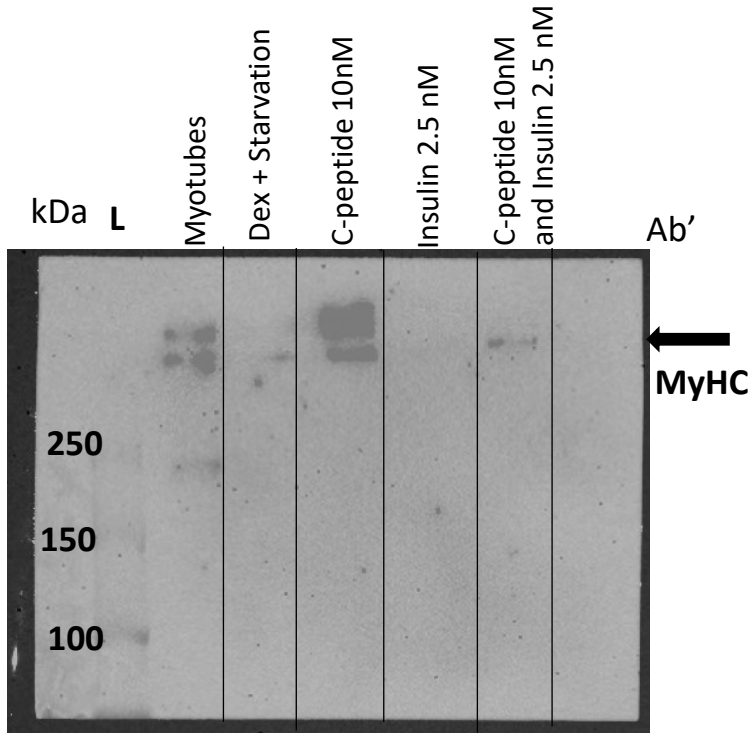

SET1

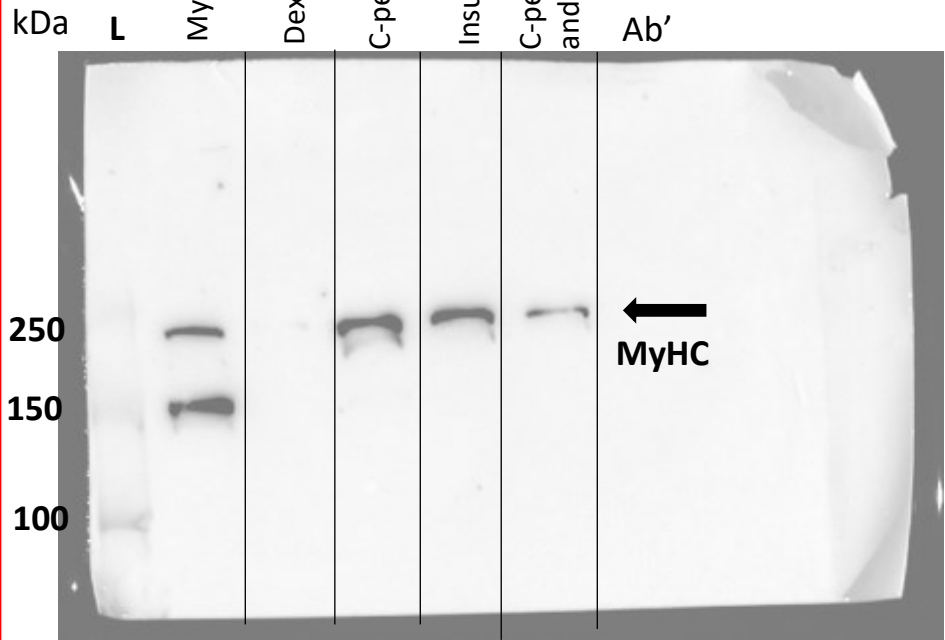

SET2

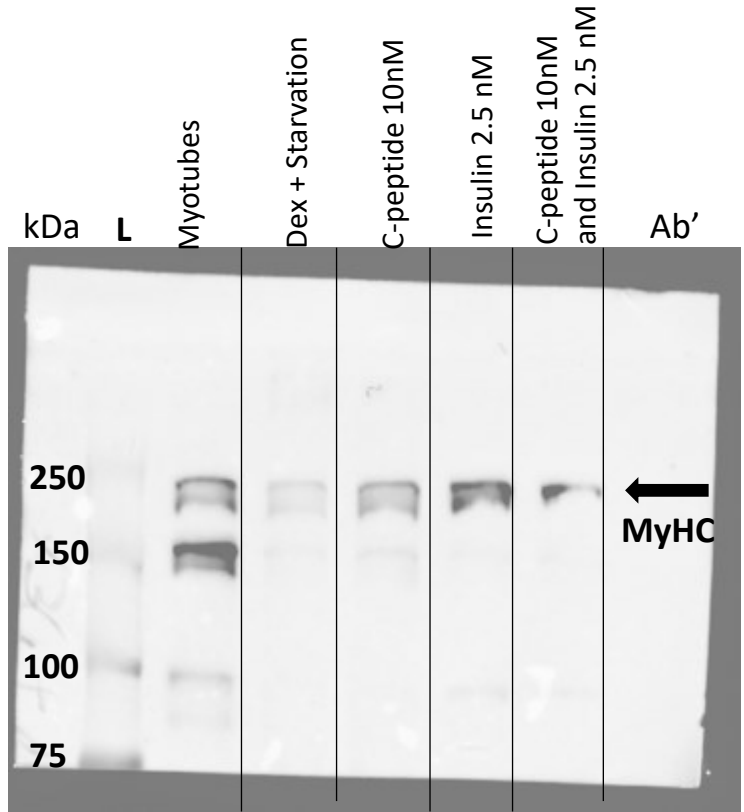

SET 3

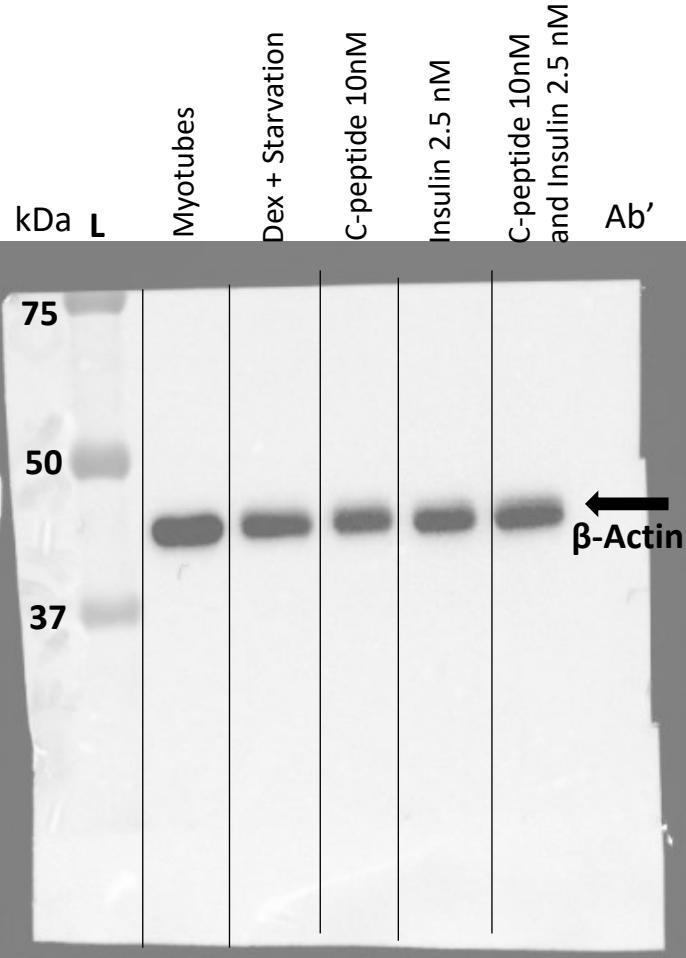

SET 1

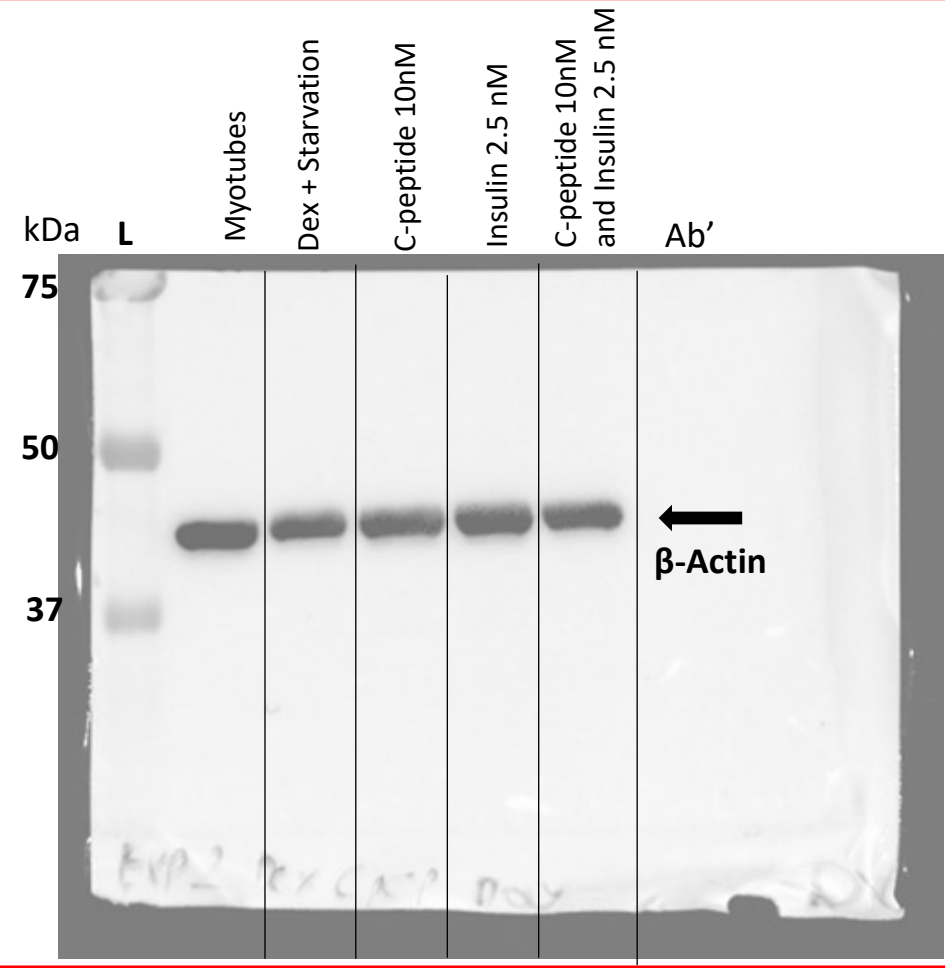

SET 2

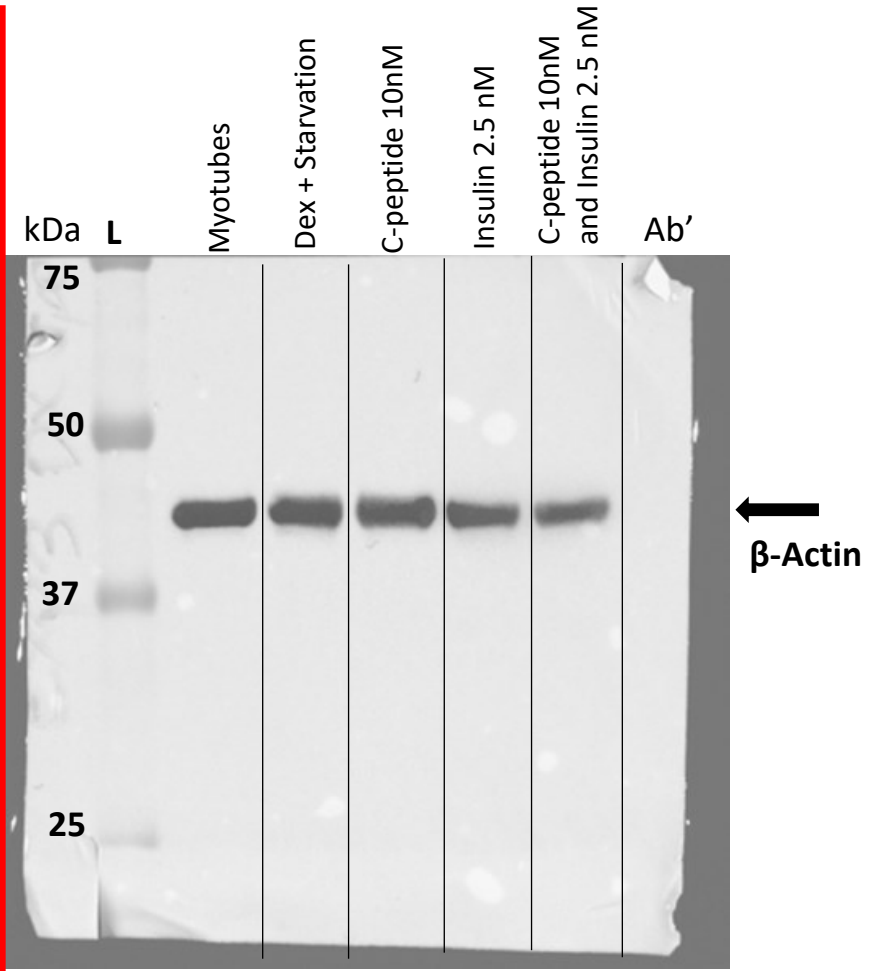

SET 3

Supplement: Supplementary file 2 — Supplementary Material 2 [file 12967_2026_7983_MOESM2_ESM.pdf]
