# Supplementary material for: C-peptide promotes myogenic differentiation in vitro and low serum levels are associated with sarcopenia in adults and the elderly
Source: J Transl Med. 2026 Mar 11;24:542. doi: 10.1186/s12967-026-07983-9 (PMC13094129; doi:10.1186/s12967-026-07983-9)
Supplement: Supplementary file 4 — Supplementary Material 4 [file 12967_2026_7983_MOESM4_ESM.docx]

**Title: C-peptide promotes myogenic differentiation in vitro and low serum levels are associated with sarcopenia in adults and the elderly**

| **Suppl.Table 1.** *Demographic, clinical and instrumental characteristics in a cohort of adults and older individuals* | | | | | | |  |
| --- | --- | --- | --- | --- | --- | --- | --- |
| Characteristics | | | Participants (N = 191) | | | |  |
| Age (years) | | | 63±8 | | | |  |
| BMI (Kg/m^2^) | | | 28.3±4 | | | |  |
| ^*^HGS (Kg) | | | 27.7±10 | | | |  |
| HGS (Kg, women) | | | 21.8±5 | | | |  |
| HGS (Kg, men) | | | 38.3±8 | | | |  |
| Glucose (mg/dL) | | | 91±11 | | | |  |
| Insulin (mU/L) | | | 12.4±7 | | | |  |
| Creatinine (mg/dL) | | | 0.80±0.2 | | | |  |
| C-peptide (ng/mL) | | | 2.08±0.9 | | | |  |
| Total Cholesterol (mg/dL) | | | 211±36 | | | |  |
| HS-CRP (mg/L) | | | 2.75±4 | | | |  |
| *Bioimpedance analysis* | | | | | | |  |
| ASMM (Kg) | | | 18.4±4 | | | |  |
| ASMM (Kg, women) | | | 16.5±3 | | | |  |
| ASMM (Kg, men) | | | 22.9±3 | | | |  |
| SMM (Kg) | | | 24.2±8 | | | |  |
| FM (%) | | | 32±7 | | | |  |
| *Prevalence* | | | | | | |  |
| Gender (Female, %) | | | 70 | | | |  |
| Smokers (%) | | | 31 | | | |  |
| MVPA (%) | | | 27 | | | |  |
| Obesity (%) | | | 34 | | | |  |
| Dyslipidemia (%) | | | 74 | | | |  |
| Lipid-lowering agents (%) | | | 16 | | | |  |
| Hypertension (%) | | | 46 | | | |  |
| Antihypertensive agents (%) | | | 43 | | | |  |
| Low ASMM/Sarcopenia (%) | | | 28 | | | |  |
| ^*^Only in 160 participants.  *BMI* body mass index, *HGS* hand grip strength, H*S-CRP* high sensitive – C reactive protein, *ASMM* appendicular skeletal muscle mass*, SMM* skeletal muscle mass, *FM* fat mass, *MVPA* moderate to vigorous physical activity.  Mean ± standard deviation. | | | | | | |  |
| **Suppl. Table 2.** *Participant’s demographic, anthropometric and clinical characteristics according to C-peptide tertiles in adults and older individuals* | | | | | | | |
| Variables | Tertile 1  (N = 63) | Tertile 2  (N = 64) | | Tertile 3  (N = 64) | *p-value* | *Bonferroni*  *Post-Hoc Analysis* | |
| C-peptide (*Range*, ng/mL) | 0.59-1.60 | 1.61-2.31 | | 2.32-4.65 |  |  | |
| Age (years) | 65±9 | 63±8 | | 60±6 | 0.002 | 1 vs 3 0.002 | |
| BMI (Kg/m^2^) | 26±4 | 28±3 | | 31±4 | <0.001 | 1 vs 2 0.009  1 vs 3 <0.001  2 vs 3 <0.001 | |
| ^*^HGS (Kg) | 25.1±8 | 28.3±9 | | 28.9±12 | 0.14 | / | |
| ASMM (Kg) | 16.3±3 | 18.8±4 | | 20.1±4 | <0.001 | 1 vs 2 0.001  1 vs 3 <0.001 | |
| ASMM (Kg, women) | 15.6±2 | 16.0±2 | | 17.7±3 | <0.001 | 1 vs 2 <0.001  1 vs 3 0.001 | |
| ASMM (Kg, men) | 21.1±3 | 23.8±2 | | 23.9±4 | 0.009 | 1 vs 2 0.008  1 vs 3 0.007 | |
| SMM (Kg) | 21.0±6 | 25.2±9 | | 26.3±8 | <0.001 | 1 vs 2 0.008  1 vs 3 0.001 | |
| FM (%) | 30±7 | 31±7 | | 35±7 | <0.001 | 1 vs 3 <0.001  2 vs 3 <0.001 | |
| Glucose (mg/dL) | 87±9 | 90±10 | | 96±11 | <0.001 | 1 vs 3 <0.001  2 vs 3 0.014 | |
| Insulin (mU/L) | 9±4 | 10±3 | | 18±8 | <0.001 | 1 vs 2 0.003  1 vs 3 <0.001  2 vs 3 <0.001 | |
| Creatinine (mg/dL) | 0.77±0.1 | 0.80±0.2 | | 0.81±0.2 | 0.41 | / | |
| Total Cholesterol (mg/dL) | 211±33 | 208±39 | | 214±36 | 0.60 | / | |
| HS-CRP (mg/dL) | 2.5±2.5 | 2.2±4 | | 3.5±5 | 0.16 | / | |
| *Prevalence* | | | | | | | |
| Gender (Female, %) | 86 | 64 | | 61 | 0.002 | / | |
| Smokers (%) | 24 | 34 | | 36 | 0.17 | / | |
| MVPA (%) | 25 | 31 | | 23 | 0.80 | / | |
| Obesity (%) | 18 | 28 | | 56 | <0.001 | / | |
| Dyslipidemia (%) | 71 | 75 | | 78 | 0.35 | / | |
| Lipid-lowering agents (%) | 11 | 20 | | 16 | 0.48 | / | |
| Hypertension (%) | 40 | 38 | | 59 | 0.031 | / | |
| Antihypertensive agents (%) | 36 | 36 | | 56 | 0.031 | / | |
| ^*^Only in 160 participants.  *BMI* body mass index, *HGS* hand grip strength, H*S-CRP* high sensitive – c reactive protein, *ASMM* appendicular skeletal muscle mass*, SMM* skeletal muscle mass, *FM* fat mass, *MVPA* moderate to vigorous physical activity.  Mean ± standard deviation. | | | | | | | |

| **Supplemental Table 3.** *Mean ± SD participants’ energy, and nutrients intake across tertiles of C-peptide in adults and older individuals* | | | | | |
| --- | --- | --- | --- | --- | --- |
| Variables | Tertile 1  (N = 63) | Tertile 2  (N = 64) | Tertile 3  (N = 64) | *p-value* | *Bonferroni*  *Post-Hoc Analysis* |
| C-peptide (*Range*, ng/mL) | 0.59-1.60 | 1.61-2.31 | 2.32-4.65 |  |  |
| Energy intake (kcal/day) | 2148±560 | 2207±579 | 2359±593 | 0.16 | / |
| Carbohydrates (%) | 44±9 | 45±8 | 46±8 | 0.47 | / |
| Proteins (%) | 14±2 | 14±3 | 14±2 | 0.88 | / |
| Fats (%) | 43±9 | 41±8 | 40±7 | 0.30 | / |
| Saturated fatty acids (g/day) | 25±10 | 26±10 | 29±13 | 0.15 | / |
| Monounsaturated fatty acids (g/day) | 59±22 | 57±21 | 58±20 | 0.91 | / |
| Polyunsaturated fatty acids (g/day) | 15±6 | 14±6 | 15±6 | 0.88 | / |
| Cholesterol (mg/day) | 237±104 | 230±92 | 256±118 | 0.40 | / |
| Fiber (g/day) | 22±6 | 23±5 | 23±7 | 0.52 | / |

Mean ± standard deviation.
