# Supplementary material for: C-peptide promotes myogenic differentiation in vitro and low serum levels are associated with sarcopenia in adults and the elderly
Source: J Transl Med. 2026 Mar 11;24:542. doi: 10.1186/s12967-026-07983-9 (PMC13094129; doi:10.1186/s12967-026-07983-9)
Supplement: Supplementary file 5 — Supplementary Material 5 [file 12967_2026_7983_MOESM5_ESM.docx]

**Supplementary Figure 1:** **HPLC Analysis of C-Peptide in Cell Culture Medium** (A) HPLC calibration curve of C-peptide prepared in DMEM cell culture medium at concentrations ranging from 0.625 to 20 nM (r² = 0.9979). (B) Quantification plot of C-peptide peak area. (C) C-peptide standard solution showing a characteristic retention time of approximately 8.6 min. (D) DMEM cell culture medium alone, demonstrating no interfering peaks at the C-peptide retention time. (E) DMEM supplemented with 10 nM C-peptide prior to incubation with cells. (F) Culture medium collected after 48 h of incubation, showing a marked reduction in C-peptide peak area compared to the pre-incubation sample.
